# Simulate the natural four-season fermentation system for high-salt diluted-state soy sauce brewing: Application in flavor promotion regulation

**DOI:** 10.1371/journal.pone.0334707

**Published:** 2025-10-16

**Authors:** Changzheng Wu, Hui Wu, Xiya Yu, Tianchang Jia, Tao He, Qinhui Hong, Xing Tong

**Affiliations:** 1 Key Laboratory of Advanced Technology Enterprise of Guangdong Seasoning Food Biofermentation, Foshan, China; 2 Guangdong Provincial Research Center of Brewing Microbiology Breeding and Fermentation Engineering Technology, Foshan, China; 3 Guangdong Haitian Innovation Technology Co., Ltd., Foshan, China; 4 Foshan Haitian (Gaoming) Flavouring & Food Co., Ltd., Foshan, China; 5 Foshan Haitian (Nanning) Flavouring & Food Co., Ltd., Nanning, China; Yantai Institute of Technology, CHINA

## Abstract

The seasonal temperature dependency of soy sauce fermentation poses significant challenges to flavor consistency, particularly under low-temperature conditions (e.g., 15°C in winter), where diminished aroma arises from microbial metabolic constraints. Traditional multi-year field studies to decipher these mechanisms are impractical for rapid industrial optimization. In this study, we present an innovative laboratory-based seasonal temperature simulation fermentation system that precisely replicates climatic conditions (15–37°C) of the Lingnan region, enabling accelerated investigation of microbial dynamics and targeted strain interventions. Our findings revealed that, compared to the 30°C (simulating the autumn season) fermented soy sauce, which exhibits optimal flavor, 15°C (simulating the winter season) had a weaker flavor of soy sauce due to the low relative abundance of specific strains, especially *Staphylococcus lloydii*, *Leuconostoc lactis*, and *Kodamaea ohmeri*. Those three strains were added into the low-temperature fermentation process, *Staphylococcus lloydii* and *Kodamaea ohmeri* promoted the formation of soy sauce key aroma compounds, such as benzene acetaldehyde, 1-octen-3-ol, and ethyl acetate. Our research offers the soy sauce industry a feasible tool to efficiently and cost-effectively test the reinoculation of functional microbial strains, thereby ensuring consistent flavor profiles throughout the year.

## Introduction

Soy sauce originated in China and has spread to Japan, South Korea, and other Asian countries, earning global popularity for its distinctive flavor and taste [1]. In traditional Chinese brewing, wheat and soybeans are the main raw materials used to produce soy sauce. The process of soy sauce fermentation involves steaming, koji making, moromi fermentation, oil pressing, and sterilization, resulting in a distinctive flavor profile [2]. Indisputably, the Lingnan region is the most famous soy sauce production area in China [3]. The region’s high-salt diluted-state soy sauce boasts exceptional quality, allowing it to command over 50% of the Chinese market [4]. Furthermore, a substantial portion of soy sauce produced in the Lingnan region is earmarked for export to international markets [5].

Moromi fermentation constitutes the most critical phase determining the flavor profile of soy sauce [6]. Throughout this stage, enzymes derived from koji persistently catalyze the degradation of macromolecular nutrients, generating flavor-enhancing amino acids, peptides, and volatile compounds [7]. During early moromi fermentation, *Weissella* and *Bacillus* genera dominate the microbial community, exhibiting robust enzymatic capabilities, including protease and amylase production. These bacteria play pivotal roles in nutrient conversion and raw material utilization, directly enhancing both flavor complexity and production efficiency [6,8]. In the mid-fermentation phase, *Tetragenococcus halophilus* emerges as the predominant halotolerant lactic acid bacteria (LAB), contributing significantly to flavor refinement and product standardization [9]. The terminal fermentation phase witnesses LAB-mediated acidification creating favorable conditions for yeast proliferation, accompanied by beneficial metabolite synthesis, including bacteriocins and γ-aminobutyric acid [10]. Ultimately, yeast predominates in the terminal phase, synthesizing compounds critical for soy sauce’s organoleptic uniqueness [11–13].

In the Lingnan region, the high-salt liquid-state (HSLS) fermentation system operates as a semi-natural ecosystem exposed to seasonal climatic variations [3]. Foshan City—the HSLS production hub in Lingnan—experiences distinct seasonal patterns: prolonged scorching summers (ambient: 30–37°C; fermentation tanks: 37°C), brief cool winters (ambient: 10–15°C; tanks: 15°C), and transient spring/autumn transitions (ambient: 20–30°C; tanks: 25–30°C). Industrial observations revealed that winter fermentation at 15°C yields sensorially inferior products compared to 30°C controls, posing significant challenges for year-round quality consistency [14]. While microbial supplementation (e.g., *Tetragenococcus halophilus* and *Wickerhamomyces anomalus*) shows potential for low-temperature (22°C) flavor enhancement [15], the underlying mechanisms remain unexplored. Temperature-driven microbial community shifts directly modulate metabolic cascades during fermentation, ultimately determining season-dependent flavor profiles [16,17]. However, the biochemical basis for aroma deficiency under low-temperature conditions persists as a critical knowledge gap. Therefore, a comprehensive investigation of temperature-microbiota-flavor interactions in natural fermentation systems is imperative to address annual product variability [16,18,19].

The current research exhibits a significant limitation: most explorations have been conducted at temperatures substantially higher than actual industrial winter conditions (15°C), creating a notable gap between existing findings and the specific solutions required to address challenges under extreme industrial low-temperature (15°C) environments. The precise biochemical mechanisms underlying impaired synthesis of flavor compounds and aroma deficiency under industrial low-temperature conditions (15°C) remain an unresolved critical scientific question. This greatly restricts the effective development and scalable application of microorganism-based targeted solutions in real production scenarios, particularly under harsh winter conditions (15°C). However, traditional seasonal mechanism investigation methods—such as multi-annual field observation studies under natural conditions—have proven inefficient due to their prolonged duration (requiring complete natural cycles), excessive resource consumption, and susceptibility to interference from uncontrollable confounding factors. This mismatch between research methodologies and industrial demands constitutes a substantial barrier to resolving the winter flavor deficiency bottleneck. To bridge this critical knowledge gap and overcome the constraints of traditional research paradigms, this study innovatively developed and implemented a laboratory-scale temperature simulation platform that precisely mimics the full temperature spectrum (15–37°C) of the Lingnan region. Validated through our preliminary work [20] for key physicochemical parameters (pH, total acidity, amino acid nitrogen kinetics) and GC-MS flavor metabolite profiles, this platform has demonstrated its capability to faithfully replicate the biochemical evolution trajectories of 200-ton industrial fermentation tanks across seasonal variations. It establishes a novel research framework for systematically investigating the mechanistic origins of low-temperature flavor deficiencies under controlled yet industrially relevant conditions.

Building upon this foundation, we employed the platform to investigate three critical objectives: (i) the influence of seasonal temperature gradients (15°C, 25°C, 30°C, 37°C) on microbial community dynamics in high-salt diluted moromi; (ii) identification and functional screening of core flavor-enhancing microbial strains; and (iii) mechanistic elucidation of how these microbial agents improve flavor profiles in low-temperature fermentation systems. This approach provides a scalable strategy to evaluate functional strain backfill effects for enhancing flavor consistency across annual production cycles.

## Materials and methods

### Strain screening for low-temperature fermentation

#### Moromi fermentation.

Soybeans (diameter 7 ± 1 mm, weight 200 ± 10 mg) and *Aspergillus oryzae* 3.042 were provided by Guangdong Haitian Innovation Technology Co., Ltd. (Foshan, China). Soybeans were first steamed and mixed with *Aspergillus oryzae* 3.042 for koji making. Subsequently, the matured koji was immersed in a saturated brine solution (m/m = 1:2) and fermented in laboratory-scale fermentation tanks for moromi fermentation with a final sodium chloride concentration of 18% (w/w) (Li et al., 2023). The fermentation temperature of moromi was separately set at 15°C (low temperature, LT), 25°C (natural temperature, NT), 30°C (middle temperature, MT), and 37°C (high temperature, HT). The whole experimental design is shown in the graphical abstract. The moromi was obtained through filtration after centrifuging samples at 10,000 rpm for 5 min, and the resulting supernatant was stored in the refrigerator at −80°C.

#### Amplicon sequencing analysis.

Total microbial DNA of samples was extracted by using the Powersoil DNA Isolation kit (MoBio, Carlsbad, CA, USA). The total DNA of samples was used as a template, and the V3-V4 region of bacteria and the ITS2 region of fungi were amplified. The construction and sequencing of PCR products were commissioned by Novogene Co., Ltd. (Beijing, China). The sequencing platform was Illumina Novaseq PE250.

#### Isolation of the microbe.

The samples were taken from fermented moromi and each sample was evenly mixed with sterile saline (w/w = 1:9) and fully shaken at 30°C for 30 min, diluted (from 1:10–1:100,000). The dilutions were spread on Luria-Bertani (LB) agar (Oxoid, Basingstoke, UK) and de Man, Rogosa and Sharpe (MRS) agar (Oxoid, Basingstoke, UK), followed by incubation at 37°C for 1–2 days to isolate bacteria. For the isolation of fungi, the dilutions were spread on Malt Extract Agar (MEA) (Oxoid, Basingstoke, UK) and incubated at 30°C for 1–2 days.

#### PCR.

The microorganisms in the samples were isolated and purified, and then the growth morphology of the colonies was observed before selecting single colonies for culture. Primers 27F (5’-AGAGTTTGATCCTGGCTCA-3’) and 1492R (5’-GGTTACCTTGTTACGACTT-3’) were used to identify the bacteria, primers ITS1 (5’-TCCGTAGGTGAACCTGCGG-3’) and ITS4 (5’-TCCTCCGCTTATTGATATGC-3’) were used to identify the fungus [21]. Each reaction was performed in a 20 μL reaction mixture containing 10 μL PrimeSTAR Max Premix (2X)* (Takara Bio (Beijing) Biotechnology Co., Ltd.), 1 μL of each primer, and 1 μL of DNA template. The PCR products were sent to BGI Co., Ltd. (Wuhan, China) for sequencing, and the sequencing results were compared with Blast homology in NCBI.

### Verification of the fermentation performance of dominant strains

According to the results of microbial diversity analysis and isolated strains, *Kodamaea ohmeri*, *Leuconostoc lactis,* and *Staphylococcus lloydii* were screened and used to verify their performance during low-temperature moromi fermentation. Koji making and brine mixing were conducted in a similar way as described in Moromi fermentation section. Moromi fermentation was separated into two control groups and three experimental groups. The moromi fermented at 15°C was set as the negative control group (group LT), while the moromi fermented at 30°C was set as the positive control group (group MT). For control groups, no other strain was inoculated into fermented moromi. For experimental groups, *Kodamaea ohmeri* (group K), *Leuconostoc lactis* (group L), and *Staphylococcus lloydii* (group S) were individually inoculated into fermented moromi with 10^7^ CFU/g bacterial suspensions. The inoculating time of *Kodamaea ohmeri* and *Staphylococcus lloydii* was the 15th day of fermentation, while the backfilling time of *Leuconostoc lactis* was the 60th day of fermentation. The fermentation temperature of three experimental groups was set at 15°C and the whole fermentation process of five groups lasted for 90 days. The whole experimental design is shown in the Graphical abstract. The moromi was obtained through filtration after centrifuging samples at 10,000 rpm for 5 min, and the resulting supernatant was stored in the refrigerator at −80°C.

#### Physicochemical characteristics analysis.

The reducing sugar (RS) content was determined using the 3,5-dinitrosalicylic acid (DNS) method. A standard curve was prepared with glucose solutions (0-1.2 mg/mL). Briefly, 10 μL of standard or a 5-fold diluted sample was mixed with 190 μL DNS reagent, vortexed, heated at 100°C for 5 min, cooled on ice, and the absorbance was measured at 540 nm using a microplate reader. The sample concentration was calculated based on the standard curve [22]. Total acid (TA) and amino nitrogen (AN) were separately determined using acid-base titration and formaldehyde titration with an automatic potentiometric titration apparatus (model 905, Metrohm, Herisau, Switzerland [22,23]. The pH value was measured using a pH meter (MettlerToledo GmbH, Greifensee, Switzerland).

#### Volatile compound analysis.

A gas chromatograph-mass spectrometer (HS-SPME-GC-MS) was used to detect volatile substances of moromi. 5 mL moromi and 25 μL solution of 2-octanol (3.35 × 10−3 mg/mL, Sigma-Aldrich, St. Louis, MO, USA) was added into a 20 mL headspace bottle (Sartorius, Goettingen, Germany). The volatile was extracted by an 85 µm carboxy/polydimethylsiloxane SPME fiber (CAR/PDMS, Supelco, Bellefonte, PA, USA) for 30 min at 40°C under 250 rpm agitation using a Combi Pal autosampler (CTC Analytics, Zwingen, Switzerland). A compound separation and analysis were performed using a GC-MS (model 7890B-5977B, Agilent, Santa Clara, CA, USA) with an HP-INOWAX capillary column (60 m length, 0.25 mm i.d., 0.25 µm film thickness, Agilent). Helium was used as the carrier gas with a flow rate of 1.2 mL/min. The oven temperature was first held at 40°C for 5 min, then increased to 240°C at a rate of 5°C/min with a holding time of 15 min.

Volatiles were identified by matching mass spectra (MS) against the NIST 17.0 and Wiley 275 databases, with additional verification via linear retention index (LRI) comparisons to literature data in the NIST WebBook. For semi-quantification, peak areas of target compounds were normalized to the internal standard (2-octanol) using the following formula:


$$\begin{array}{cc} Concentration\;\left(\mu g/L\right)= & \frac{Peak\;Area\;of\;Target\;Compound}{Peak\;Area\;of\;\;2\;-\;octanol}\times \frac{Concentration\;of\;\;2\;\;-\;octanol\;\left(\mu g/mL\right)}{Volume\;of\;Moromi\;Sample\;\left(mL\right)}\\ & \times \frac{Volume\;of\;Internal\;Standard\;\left(mL\right)}{Volume\;of\;Moromi\;Sample\;\left(mL\right)} \end{array}$$ (1)

This approach accounts for variations in extraction efficiency and instrumental response. To ensure accuracy, recovery experiments were conducted by spiking known amounts of 2-octanol into moromi samples (n = 6), yielding average recoveries of 92–108% with relative standard deviation (RSD) < 5%.

All volatile compound concentrations were reported as mean values ± standard deviation (n = 3). Additionally, inter-day and intra-day precision were evaluated by analyzing replicate samples (n = 6) across three independent experiments, with RSD values for peak areas < 10% for all target compounds. This comprehensive validation ensures the reliability of semi-quantitative results under the described experimental conditions.

### Statistical analysis

All data were analyzed using IBM SPSS 24.0 (SPSS Inc., Chicago, IL, USA). Based on 10 dominant bacterial genera and 8 dominant fungal genera, LEfSe comparison and Spearman correlation analysis were performed using the online platform of BGI (Wuhan, China). Microbial markers with LDA > 3.5 were screened to determine the characteristic bacteria of different samples. Pearson and Spearman correlation analysis was conducted with 4 physicochemical factors (reducing sugar, total nitrogen, amino acid nitrogen, and total acid), 17 free amino acids, and 9 volatile flavor substances that have been measured and published [20]. GraphPad Prism 9.4.1 (GraphPad Software, San Diego, CA, USA) was used to draw line charts and bar charts. The correlation analysis was carried out using R software (version R-4.2.2) with package psych and the orthogonal partial least squares discrimination analysis (OPLS-DA) was carried out with SIMCA 14.1 (Umertrics, Sweden). A significance level of p < 0.05 was chosen.

## Results and discussion

### Seasonal temperature profile in industrial-scale fermentation

Prior to conducting laboratory-scale experiments, we monitored soy sauce fermentation temperatures in three 200-ton industrial-scale fermentation tanks located in Foshan, China, from 2020 to 2022. Statistical analyses revealed distinct seasonal patterns (Table 1). The lowest average temperature (15.4 ± 0.8°C) was consistently observed during the winter months (December to February). During the transitional periods of spring (March to May) and autumn (September to November), temperature fluctuations were significantly more pronounced. The mean fermentation temperatures were 25.3 ± 2.4°C in spring and 29.7 ± 3.2°C in autumn, reflecting distinct seasonal averages with associated variability. The highest fermentation temperature occurred in summer (June–August), with the moromi reaching an average temperature of 37.1 ± 1.4°C. These empirical findings provide a robust foundation for our laboratory temperature settings: LT (15°C) simulates winter fermentation conditions, NT (25°C) and MT (30°C) represent the mean temperatures observed during spring and autumn fermentation, respectively, while HT (37°C) corresponds to the peak temperatures encountered in summer under actual production conditions.

**Table 1:** Seasonal variation of average moromi temperature in industrial-scale fermentation (200-ton tanks), between 2020-2022.

Year		2020	2021	2022	Monthly Average Temperature	Seasonal Average Temperature
Spring Temperature inside fermentation tanks (°C)	March	23.1	23.9	22.9	23.3 ± 0.4	25.3 ± 2.4
	April	24.5	24.1	23.6	24.1 ± 0.4	
	May	28.7	29.4	27.9	28.7 ± 0.6	
Summer Temperature inside fermentation tanks (°C)	June	35.7	34.5	35.4	35.2 ± 0.5	37.1 ± 1.4
	July	38.6	38.6	38.8	38.7 ± 0.1	
	August	37.9	37.5	37.3	37.6 ± 0.2	
Autumn Temperature inside fermentation tanks (°C)	September	34.5	33.4	33.3	33.7 ± 0.5	29.7 ± 3.2
	October	29.8	28.5	29.6	29.3 ± 0.6	
	November	25.3	26.3	26.4	26.0 ± 0.5	
Winter Temperature inside fermentation tanks (°C)	December	13.9	16.4	13.9	14.7 ± 1.2	15.4 ± 0.8
	January	14.6	14.6	15.9	15.0 ± 0.6	
	February	18.0	19.5	12.1	16.5 ± 3.2	

#### Microbial diversities.

Amplicon sequencing revealed distinct distribution patterns of bacterial and fungal communities in moromi under varying fermentation temperatures, demonstrating differential microbial community structures among fermentation groups. Alpha diversity indices (Table 2) quantified microbial richness and diversity. Fungal community analysis showed notably elevated Chao1 indices (>200) in both HT and LT groups. The HT group exhibited peak Chao1 (222.74) and Shannon (2.37) indices on day 15, whereas the NT group reached maximum values (Chao1: 222.47; Shannon: 2.39) by day 60, suggesting sustained fungal ecosystem richness in these experimental groups. In contrast, bacterial communities displayed significant fluctuations in both Chao1 and Shannon indices across all four groups, indicative of dynamic compositional changes during fermentation.

**Table 2:** Comparison of the diversity index of bacteria and fungi communities between group LT, group NT, group MT, and group HT (HT: 37°C, MT: 30°C, NT: 25°C, LT: 15°C).

Group	Time of fermentation (days)	Bacteria		Fungi	
		Chao1	Shannon	Chao1	Shannon
HT	5	480.95	3.22	148.72	1.49
	15	486.42	3.27	222.74	2.37
	25	651.80	4.02	119.57	1.53
	35	244.10	3.86	56.25	0.86
	60	802.09	5.47	67.69	0.92
MT	5	535.14	3.88	84.42	1.64
	15	333.74	2.95	46.16	1.04
	25	343.84	2.99	113.03	1.86
	35	514.77	4.11	51.17	1.5
	60	485.19	4.02	57.89	1.48
LT	5	555.44	2.77	16.5	0.12
	15	857.67	3.45	53.62	1.23
	25	469.66	2.28	99.86	1.59
	35	264.92	3.25	143.79	2.26
	60	671.94	4.82	114.19	1.66
NT	5	510.19	2.88	92.69	2
	15	314.87	2.86	62.85	1.42
	25	658.48	4.20	107.25	1.34
	35	725.49	4.69	130.67	1.55
	60	595.08	5.16	222.47	2.39

#### Influence of fermentation temperature on microbial succession.

**Fig 1:**
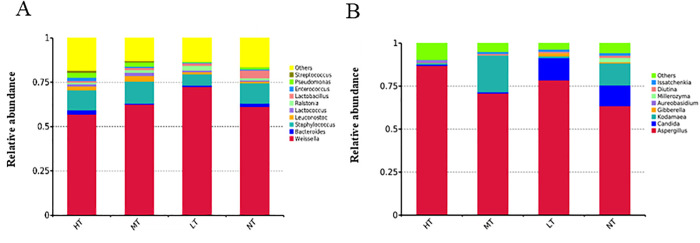
Microbial community composition in moromi under different fermentation temperatures (HT: 37°C, MT: 30°C, NT: 25°C, LT: 15°C). **(A)** Bacterial community; **(B)** Fungal community.

Ten dominant bacteria genera (Fig 1A) and 8 dominant fungal genera (Fig 1B) were observed with relative abundance greater than 1%. In Fig 1A, *Weissella* and *Staphylococcus* were the dominant bacterial genera with the highest relative abundance (the mean relative abundances were 63.36% and 10.41%) in all four groups. Eight dominant bacteria genera were observed in group HT (*Weissella*, *Leuconostoc*, *Lactococcus*, *Enterococcus*, *Staphylococcus*, *Bacteroides*, *Pseudomonas,* and *Streptococcus*), *Enterococcus* and *Streptococcus* existed only in group HT. Seven dominant bacteria genera (*Weissella*, *Staphylococcus*, *Leuconostoc*, *Pseudomonas*, *Lactococcus*, *Ralstonia,* and *Lactobacillus*) were found in group MT and *Weissella* has the highest relative abundance (62.56%). This was consistent with the findings of Liang et al. [24], suggesting that temperature-controlled fermentation (30°C) boosted the growth of *Weissella* in soy sauce compared with fermentated at 25°C. Six dominant bacteria genera (*Weissella*, *Bacteroides*, *Staphylococcus*, *Ralstonia*, *Lactobacillus* and *Pseudomonas*) were found in group NT. The relative abundance of *Lactobacillus* (4.54%) in group NT was higher than that of the other three groups. Six dominant bacteria genera (*Weissella*, *Bacteroides*, *Staphylococcus*, *Ralstonia*, *Lactobacillus* and *Pseudomonas*) were found in group NT. The relative abundance of *Lactobacillus* (4.54%) in group NT was higher than that of the other three groups. Five dominant bacteria genera (*Weissella*, *Staphylococcus*, *Leuconostoc*, *Lactobacillus* and *Ralstonia*) were found in group LT. The relative abundance of *Weissella* in group LT reached 72.50% and was higher than the other three groups (group NT: 61.28%, group HT: 56.96% and group MT: 62.56%). This was possibly due to the fact that low-temperature fermentation was more conducive to the growth of *Weissella* [24]. In contrast, the relative abundance of *Staphylococcus* was the lowest in group LT (6.34%) compared with the other three groups (group NT: 11.40%; group HT: 11.36% and group MT: 12.51%). Nguyen et al. [25] reported that low-temperature fermentation can decrease the relative abundance of *Staphylococcus* in moromi. It was reported that *Weissella* and *Tetrastreptococcus* were more abundant in Japanese-style soy sauce (25°C) while *Staphylococcus* was more abundant in Cantonese-style soy sauce (18–22°C) [26], suggesting the temperature has different driving effects on microbial growth.

In Fig 1B, six types of dominant fungal genera were found in group NT, including *Aspergillus*, *Candida*, *Kodamaea*, *Millerozyma*, *Diutina* and *Issatchenkia*. *Aspergillus* and *Aureobasidium* were the dominant fungal genera in group HT, while *Aspergillus*, *Kodaomyces* and *Issatchenkia* were the dominant fungal genus in group MT. *Aspergillus*, *Candida*, *Kodamaea*, *Gibberella,* and *Issatchenkia* were the dominant fungal genus in group LT, and *Gibberella* was only found in the group LT. *Aspergillus* was the dominant fungal genera in all four groups since *Aspergillus oryzae* was used as the start strain for koji making [26]. The relative abundance of *Aspergillus* in group HT reached 86.86% and was much higher than that in group MT (70.83%), group NT (63.49%), and group LT (78.47%), respectively. The relative abundance of *Candida* in group HT (0.79%) and group MT (0.79%) was smaller than that in group NT (12.05%) and group LT (12.96%). This can be inferred that low-temperature fermentation was more conducive to the growth of *Candida*.

### Influence of fermentation time on microbial succession

**Fig 2:**
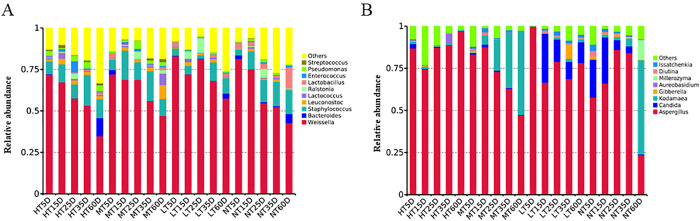
Microbial community abundance in moromi under different fermentation temperatures (HT: 37°C, MT: 30°C, NT: 25°C, LT: 15°C). **(A)** Bacterial community; **(B)** Fungal community.

During the early fermentation period (0–15 days), *Weissella* and *Staphylococcus* were the dominant bacteria in all four groups (Fig 2A). This was consistent with the findings of previous studies, showing that *Weissella* and *Staphylococcus* were dominant microorganisms during the first 15 days of soy sauce fermentation [9,27]. On the 5th day of fermentation, the relative abundance of *Pseudomonas* in group HT was 2.98% and was higher than that of the other three groups (group NT: 0.26%, group MT: 0.97% and group LT: 0.40%). Zhou et al. [4] reported that *Pseudomonas* metabolized arginine to produce ethyl carbamate through the arginine deimide pathway, reducing the content of arginine in moromi samples during fermentation. The average relative abundance of *Pseudomonas* in group HT, group MT, group NT, and group LT were 2.89%, 2.27%, 1.05% and 0.61%, respectively. This suggested that a higher fermentation temperature can result in a higher abundance of *Pseudomonas*. The relative abundance of *Pseudomonas* in group MT increased firstly from the 0th day to the 25th day and reached the highest (4.86%), and subsequently decreased with the increase of fermentation time. The relative abundance (from the 5th day to the 15th day) of *Leuconostoc*, *Lactococcus,* and *Ralstonia* in group LT increased by 1.72%, 1.64% and 4.35%, respectively. On the 60th day of fermentation, the relative abundance of *Lactobacillus* in group NT (12.83%) was higher than that of the other three groups (group HT: 1.48%, group MT: 1.80% and group LT: 1.94%). On the 60th day of fermentation, the relative abundance of *Bacteroides* in all four groups reached the highest (group HT: 10.98%, group MT: 0.06%, group NT: 5.49%, group LT: 2.97%). It was speculated that high temperature was more conducive to the growth of *Bacteroides*.

For fungi (Fig 2B), the relative abundance of *Aspergillus* in group HT was maintained at a high level throughout the whole fermentation process, reaching the highest (96.9%) on day 60. In group MT, the abundance of *Kodamaea* gradually increased from 5.37% on the 15th day to 49.55% on the 60th day, the relative abundance of *Kodamaea* (49.55%) was higher than that of *Aspergillus* (47.22%). In group MT and group NT, the relative abundance of *Kodamaea* increased with the increase of fermentation time; however, the abundance of *Kodamaea* in group LT was low and reached the highest (1.82%) on the 35th day of fermentation. On the 60th day of fermentation, the relative abundance of *Kodamaea* in group NT reached the highest (55.86%) and was higher than the other three groups (group HT: 0.55%; group MT: 49.55%; group LT: 1.68%). The abundance of *Candida* in group LT decreased significantly from 29.01% on the 15th day to 12.35% on the 60th day. He et al [28]suggested that *Candida* improved the flavor composition of *Aspergillus*-type moromi and significantly increased the content of amino acids, organic acids, and unsaturated fatty acids. Overall, among all four groups, *Weissella* and *Staphylococcus* were the dominant bacterial genera during the whole fermentation, while *Aspergillus*, *Kodamaea,* and *Candida* were the dominant fungal genera.

### Analysis of significant differences between microbiomes

**Fig 3:**
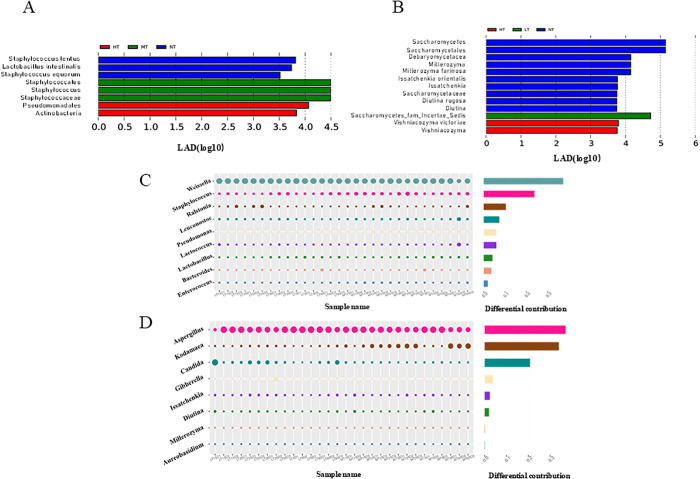
Microbial community analysis of moromi under different temperature conditions (HT: 37°C, MT: 30°C, NT: 25°C, LT: 15°C). **(A)** Bacterial biomarkers (LEfSe); **(B)** Fungal biomarkers (LEfSe); **(C)** Bacterial community differences (SIMPER, MT vs LT); (D) fungal community differences (SIMPER, MT vs LT).

As illustrated in Fig 3A and 3B, the biomarkers in group NT were *Staphylococcus lentus*, *Staphylococcus equorum*, *Lactobacillus reuteri*, *Debaryomycetaceae*, *Millerozyma*, *Diutina,* and *Issatchenkia*. The biomarkers in group HT were *Actinobacteria*, *Pseudomonadales,* and *Vishniacozyma*. The biomarker of group MT was *Staphylococcus*. The biomarker of group LT was *Saccharomycetes_fam_Incertae_Sedis*.

As shown in Fig 3C and 3D, throughout the fermentation process of groups MT and LT, the contribution of different dominant bacteria was *Weissella* (0.32), *Staphylococcus* (0.15), *Ralstonia* (0.07), *Leuconostoc* (0.05), *Lactococcus* (0.04), *Pseudomonas* (0.04), *Lactobacillus* (0.03), and *Bacteroides* (0.02). The contributions of dominant fungi were thoroughly examined as well, including *Aspergillus* (0.39), *Kodamaea* (0.31), *Candida* (0.17), *Gibberella* (0.03), *Issatchenkia* (0.02), *Diutina* (0.02), *Millerozyma* (0.01), and *Aureobasidium* (0.01) in descending order.

### Potential functions of dominant microorganisms

**Fig 4:**
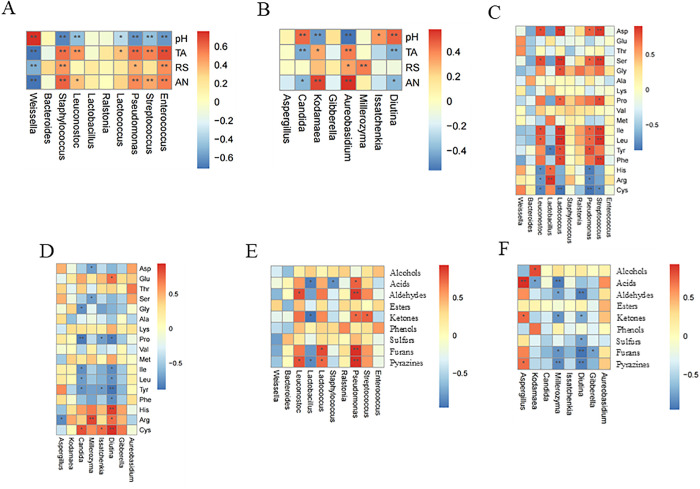
Spearman correlation analysis between dominant microorganisms and various indices in moromi. **(A)** Bacteria vs physicochemical factors; **(B)** Fungi vs physicochemical factors; **(C)** Bacteria vs free amino acids; **(D)** Fungi vs free amino acids; **(E)** Bacteria vs flavor indices; **(F)** Fungi vs flavor indices.

As shown in Fig 4A and 4B, seven bacterial genera (*Weissella*, *Staphylococcus*, *Leuconostoc*, *Lactococcus*, *Streptococcus*, *Pseudomonas,* and *Enterococcus*) and six fungal genera (*Kodamaea*, *Candida*, *Aureobasidium*, *Millerozyma*, *Diutina,* and *Issatchenkia*) showed significant positive correlation with pH and the content of TA, RS, and AN (p* *< 0.05).

The TA was significantly positively correlated with *Staphylococcus*, *Leuconostoc*, *Lactococcus*, *Streptococcus*, *Pseudomonas*, *Enterococcus*, *Kodamaea,* and *Aureobasidium* (p < 0.05). *Staphylococcus*, *Pseudomonas*, *Enterococcus*, *Aureobasidium,* and *Millerozyma* showed a significant positive correlation with RS (p < 0.05). A significant negative correlation was observed between *Weissella* and RS (p < 0.01), indicating that *Weissella* metabolism may consume a large amount of RS.

AN was significantly positively correlated with *Staphylococcus*, *Leuconostoc*, *Streptococcus*, *Pseudomonas*, *Enterococcus*, *Kodamaea,* and *Aureobasidium* (p < 0.05). This showed that *Staphylococcus*, *Kodamaea,* and *Pseudomonas* could promote the formation of AN in moromi. *Staphylococcus* can secrete enzymes related to proteolytic hydrolysis and hydrolyze soybean protein into amino acids and small molecular peptides in the process of soy sauce fermentation, thereby increasing the content of AN in moromi [26,29].

In Fig 4C–4F, *Leuconostoc*, *Lactococcus*, *Lactobacillus*, *Streptococcus,* and *Pseudomonas* were significantly correlated with 11 free amino acids and 5 flavor substances (p < 0.05). *Aspergillus*, *Candida*, *Millerozyma*, *Issatchenkia,* and *Diutina* were significantly correlated with 12 free amino acids (p < 0.05). *Aspergillus*, *Kodamaea*, *Millerozyma*, *Diutina,* and *Gibberella* were significantly correlated with the 7 flavor compounds (p < 0.05). *Leuconostoc* and *Pseudomonas* had a positive correlation with Asparagine (Asp), Serine (Ser), Leucine (Leu), and other umami and sweet amino acids (p < 0.05). *Leuconostoc* and *Aspergillus* were reported to contribute to the formation of aroma and flavor compounds [5].

Specific bacterial genera such as *Leuconostoc*, *Lactococcus*, *Ralstonia*, *Streptococcus,* and *Pseudomonas* are closely related to the production of flavor compounds (Fig 4E). *Leuconostoc* and *Lactococcus* displayed a positive relationship with aldehydes and pyrazines, while *Lactobacillus* showed a negative correlation with acid esters, aldehydes, ketones, and pyrazines. Five dominant yeast strains have a positive relationship with alcohols, esters, and phenolic substances, with *Kodamaea* demonstrating a particularly strong correlation with phenolic substances (Fig 4F), which may be related to the strong ability of yeast to produce aroma [29].

### Selection of dominant microorganisms

Eight bacterial and five fungal species were isolated from four sample types, including *Staphylococcus gallinarum*, *Enterococcus faecalis*, *Weissella sinusophagus*, *Staphylococcus lloydii*, *Enterococcus lactate*, *Staphylococcus saccharolyticus*, *Leuconostoc lactis*, *Pediococcus pentosaceus*, *Kodamaea ohmeri*, *Clavispora lusitaniae*, *Candida tropicalis*, *Millerozyma farinosa*, and *Candida orthopsilosis*. Key contributors between the HT and LT groups were bacterial genera *Weissella* (0.32), *Staphylococcus* (0.15), *Ralstonia* (0.07), and *Leuconostoc* (0.05), and fungal genera *Aspergillus* (0.39) and *Kodamaea* (0.31). Given the high abundance of *Weissella* and *Aspergillus* in group LT (*Weissella*: 72.50%, *Aspergillus*: 78.47%), their addition during low-temperature fermentation was unnecessary. *Ralstonia* could not be isolated from moromi, likely due to the culture temperature (15°C to 37°C) being unsuitable for its growth. *Staphylococcus lloydii* (*S. lloydii*) and *Kodamaea ohmeri* (*K. ohmeri*) were isolated from group MT. *Staphylococcus* enhances flavor ester production in fermented foods, correlating with RS and TA content [30]. *Kodamaea ohmeri* contributes to aroma and flavor compound formation [31–33]. *Leuconostoc lactis* (*L. lactis*), isolated from group LT, decomposes carbohydrates into alcohols and organic acids, enriching food aroma [34]. These three strains were selected as representative strains for their genera.

On fermentation day 15, group LT showed a lower relative abundance of *Staphylococcus* and *Kodamaea* compared to group MT. *Leuconostoc*’s abundance was lowest in group LT on day 60. Consequently, *Staphylococcus* and *Kodamaea* were backfilled on day 15 (groups S and K), and *Leuconostoc* on day 60 (group L) in group LT.

**Fig 5:**
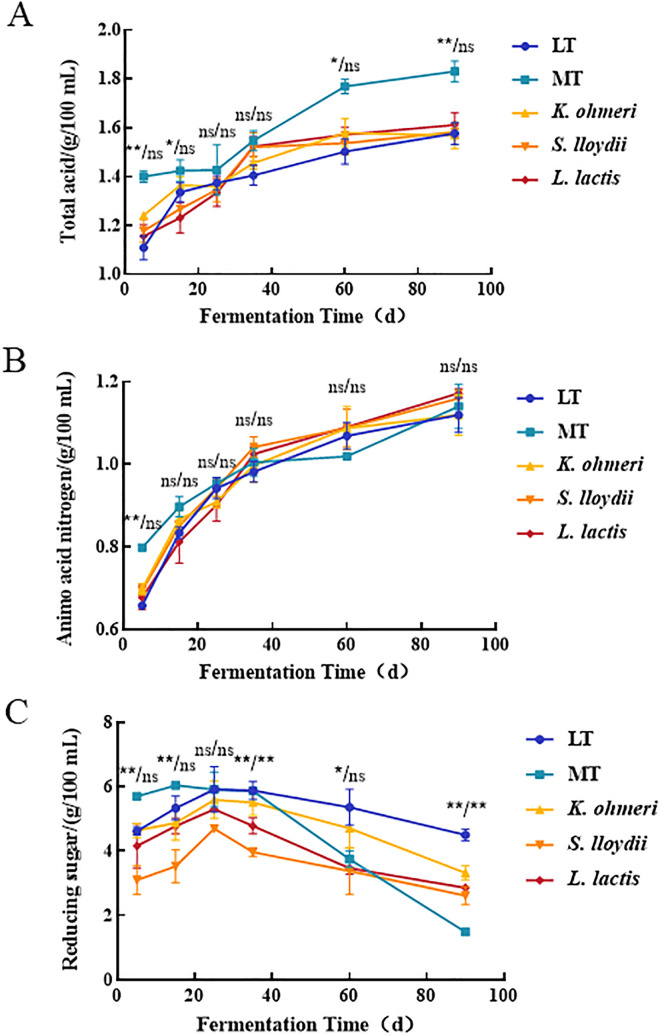
Changes of physicochemical indexes in five groups of moromi during fermentation. **(A)** Changes of total acid; **(B)** Changes of amino acid nitrogen; **(C)** Changes of reducing sugar. (MT: 30°C; LT: 15°C; *S. lloydii*: inoculated *S. lloydii*, 15°C; *L. lactis*: inoculated *L. lactis*, 15°C; *K. ohmeri*: inoculated *K. ohmeri*, 15°C). Note: *, **, ns denote the significance analysis between different samples at the same fermentation time; * indicates p < 0.05, ** indicates p < 0.01, and ns indicates not significant. The left side of the “/” indicates the significance of all five groups of samples; the right side of the “/” indicates the significance of the other four groups of samples except the group MT.

By day 90, TA and AN content in groups S and K were slightly higher than in group LT (Fig 5A and 5B), but differences were insignificant (p > 0.05). AN content across groups MT, LT, S, L, and K was 1.14, 1.19, 1.16, 1.17, and 1.17 g/100 mL, all exceeding China’s premium-grade soy sauce standard (0.80 g/100 mL, GB/T 18186−2000). The Maillard reaction slowly consumed amino acids and AN at low temperatures [15], leading to higher AN content in low-temperature groups (L, S, K) than in group MT, with no significant difference (p > 0.05). This aligns with Liu et al. [22], indicating no significant AN difference despite added microbes. Microorganisms secreted amylase to degrade bean starch, causing RS levels to rise rapidly in the first 25 days (Fig 5C). RS content on day 25 was highest in group LT (5.91 g/100 mL), followed by groups K, L, S, and MT. Inoculating *S. lloydii*, *K. ohmeri*, and *L. lactis* improved RS utilization, reducing RS content in groups S, K, and L (2.61, 3.35, and 2.85 g/100 mL) compared to group LT on day 90. This confirms prior research showing microbial addition enhances RS utilization [9].

### Volatile compound analysis

**Fig 6:**
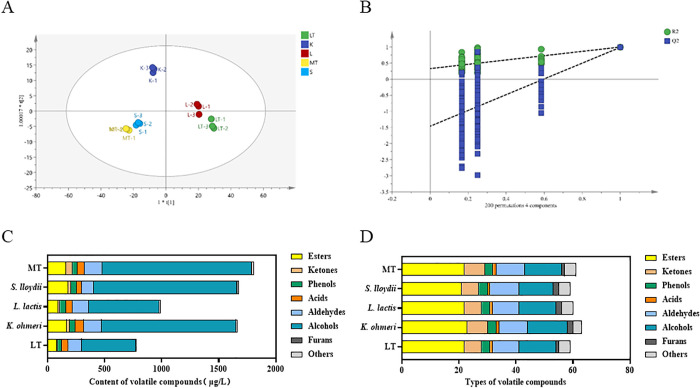
Volatile compound profiles of moromi under different fermentation conditions. **(A)** OPLS-DA score plot; **(B)** Permutation test of the OPLS-DA model; **(C)** Content of volatile compounds; **(D)** Types of volatile compounds. (MT: 30°C; LT: 15°C; *S. lloydii*: inoculated *S. lloydii*, 15°C; *L. lactis*: inoculated *L. lactis*, 15°C; *K. ohmeri*: inoculated *K. ohmeri*, 15°C).

Compared to group LT, inoculating the three strains increased volatile compound production (Fig 6A). Volatile compound content in low-temperature fermentation moromi rose (group LT: 780.73 μg/L, group K: 1661.74 μg/L, group L: 987.37 μg/L, group S: 1676.01 μg/L) but remained lower than in higher-temperature fermentation (group MT: 1810.00 μg/L). As shown in Table 2, the main volatile compounds in the fermented moromi of the two control groups and three experimental groups were classified into alcohols, esters, aldehydes, and phenols. Alcohols, key flavor compounds in soy sauce, can provide sweet and nutty flavors and are formed during the Maillard reaction [14,33]. The alcohol content in groups K, L, and S increased significantly compared to group LT (2.48 × , 1.30 × , and 2.64 × , respectively). Consistent with Li et al. [15], ethanol was the dominant compound in all fermentation groups. Notably, ethanol content in groups K and S increased 2.78-fold and 2.87-fold compared to group LT. Ethanol, primarily produced by yeast anaerobic fermentation [35], is the main source of soy sauce’s ‘alcohol flavor’ [8]. This aligns with Zhao et al. [36], who found that *Kodamaea* promotes alcohol production in fermentation systems. In this study, adding *K. ohmeri* and *S. lloydii* boosted ethanol production in groups S and K.

The OPLS-DA model demonstrated that volatile compounds in the two control and three experimental groups could be clearly differentiated (Fig 6A and 6B). As shown in Fig 6C and 6D, groups MT, LT, K, S, and L contained 61, 59, 63, 59, and 60 types of volatile compounds, respectively. This indicates that inoculating *L. lactis* and *S. lloydii* increased both the variety and content of volatile compounds in moromi. The key volatile compounds in the five groups were categorized into alcohols, esters, aldehydes, and furans (Table 3). Eight compounds with OAV > 1.0 were identified, including 3-methyl-1-butanol, 1-octen-3-ol, ethyl 3-methylbutanoate, ethyl acetate, 2-methylbutyraldehyde, 3-methylbutyraldehyde, benzene acetaldehyde, and HEMF, which distinguished the aroma characteristics of the groups. 1-octen-3-ol, a product of lipid oxidation, has a low odor threshold (1.50 μg/L) and imparts mushroom and floral notes. It is common in fermented foods [37]. The OAV of 1-octen-3-ol was higher in groups K, L, and S than in groups LT and MT, suggesting that adding *K. ohmeri*, *S. lloydii*, and *L. lactis* promoted 1-octen-3-ol production, with low temperatures favoring its formation in moromi.

**Table 3:** The key volatile compounds of five groups of moromi in the 90th day of fermentation (MT: 30°C; LT: 15°C; *S. lloydii*: inoculated *S. lloydii*, 15°C; *L. lactis*: inoculated *L. lactis*, 15°C; *K. ohmeri*: inoculated *K. ohmeri*, 15°C).

RI ^a^	Volatile Compounds	Odor threshold(μg/L)	Content (μg/L)					Odor activity value (OAV)				
			LT	MT	*K. ohmeri*	*L. lactis*	*S. lloydii*	LT	MT	*K. ohmeri*	*L. lactis*	*S. lloydii*
	**Alcohol**											
912	Ethanol	100000	361.23 ± 25.04 b	1087.08 ± 52.73 a	1005.15 ± 11.15 a	493.18 ± 15.01 b	1037.58 ± 33.62 a	<1	<1	<1	<1	<1
1184	3-Methyl-1-butanol	0.29	40.48 ± 6.85 b	83.17 ± 14.36 a	57.26 ± 8.91 b	33.35 ± 7.43 b	74.80 ± 21.37 a	139.09	285.80	196.75	114.59	257.04
1439	1-Octen-3-ol	1.5	36.24 ± 4.41 a	14.58 ± 2.57 b	47.15 ± 0.001 a	39.19 ± 0.010 a	65.20 ± 0.003 a	24.16	9.72	31.43	26.13	43.47
1708	1-Propanol	500	3.29 ± 0.24 b	17.81 ± 2.55 a	5.40 ± 0.94 b	5.62 ± 1.31 b	5.69 ± 1.01 b	<1	<1	<1	<1	<1
1732	Phenylethyl alcohol	390	7.85 ± 2.21 c	65.67 ± 8.99 a	16.05 ± 2.05 c	12.47 ± 3.84 c	25.99 ± 6.97 b	<1	<1	<1	<1	<1
1971	Maltol	2500	2.72 ± 0.07 b	9.53 ± 1.86 a	6.99 ± 0.97 a	5.23 ± 1.81 a	4.86 ± 1.52 a	<1	<1	<1	<1	<1
	**Esters**											
883	Ethyl acetate	5	40.53 ± 6.28 b	104.43 ± 21.73 a	96.88 ± 18.46 a	45.84 ± 9.83 b	113.45 ± 18.11 a	8.11	20.89	19.38	9.17	22.69
1027	Ethyl 3-methylbutanoate	0.3	0.96 ± 0.04 b	2.15 ± 0.48 a	2.72 ± 0.33 a	1.48 ± 0.19 b	3.56 ± 0.74 a	3.20	7.16	9.06	4.94	11.87
2247	Ethyl palmitate	2000	5.31 ± 1.03 b	17.33 ± 3.61 a	19.74 ± 4.11 a	8.10 ± 0.30 b	23.23 ± 2.54 a	<1	<1	<1	<1	<1
2523	Ethyl linoleate	30943	1.91 ± 0.03 b	7.65 ± 0.94 a	4.81 ± 0.20 a	2.76 ± 0.09 b	6.84 ± 1.13 a	<1	<1	<1	<1	<1
1671	Ethyl benzoate	53	1.41 ± 0.11 b	8.89 ± 2.16 a	5.40 ± 1.37 a	2.23 ± 0.84 b	6.62 ± 0.97 a	<1	<1	<1	<1	<1
1785	Ethyl phenylacetate	155	0.41 ± 0.01 c	3.96 ± 0.56 a	1.80 ± 0.51 b	0.73 ± 0.01 c	2.38 ± 0.73 b	<1	<1	<1	<1	<1
	**Aldehydes**											
911	2-Methylbutyraldehyde	4.4	34.61 ± 5.21 a	37.45 ± 4.98 a	37.70 ± 6.84 a	43.63 ± 9.43 a	24.63 ± 3.77 b	7.87	8.51	8.57	9.83	5.60
916	3-Methylbutyraldehyde	1.2	49.49 ± 10.35 a	56.82 ± 9.16 a	59.39 ± 6.77 a	57.62 ± 8.46 a	36.17 ± 3.44 b	41.24	47.35	49.49	48.02	30.14
1440	3-Furaldehyde	3000	2.68 ± 0.31 a	3.09 ± 0.95 a	5.68 ± 1.03 a	4.04 ± 0.81 a	3.93 ± 0.75 a	<1	<1	<1	<1	<1
1530	Benzaldehyde	350	13.82 ± 1.60 b	29.77 ± 5.18 a	25.10 ± 4.41 a	18.02 ± 4.54 a	21.55 ± 4.19 a	<1	<1	<1	<1	<1
1619	Benzene acetaldehyde	4	8.58 ± 1.42 a	20.29 ± 4.28 a	13.82 ± 3.21 a	11.74 ± 3.48 a	9.62 ± 2.89 a	2.15	5.07	3.46	2.94	2.41
	**Furanone**											
1090	HEMF^b^	20	ND^c^	50.23 ± 10.16 a	16.42 ± 4.71 b	5.82 ± 1.06 c	19.78 ± 5.48 b	ND	2.51	<1	<1	<1

^a^: Retention Index (RI);

^b^: 4-Hydroxy-2(or 5)-ethyl-5(or 2)-methyl-3(2H)-furanone;

^c^: Not found.

Different letters in the same row indicate significant differences at p ≤ 0.05. Values are the mean±standard deviation of three independent replicates (n = 3).

Benzaldehyde and benzene acetaldehyde, derived from phenylalanine degradation, impart almond and sweet flavors and are key aroma components [38]. The OAV of benzene acetaldehyde was higher in groups K (3.46), L (2.94), and S (2.41) than in group LT (2.15), indicating increased benzene acetaldehyde content in moromi after inoculating *K. ohmeri*, *L. lactis*, and *S. lloydii*. Although benzaldehyde was not a key flavor component in this study, its low odor threshold (4.00 μg/L) contributes significantly to fermented soy sauce flavor [15]. The OAV of 2-methylbutanal and 3-methylbutanal was higher in group MT (8.51 and 47.35, respectively) than in group LT (7.87 and 41.24, respectively), consistent with [14], who reported higher 2-methylbutanal and 3-methylbutanal content in 30°C-fermented moromi than in low-temperature-fermented moromi. Yeast and lactic acid bacteria can induce Strecker degradation of amino acids to form these compounds [37]. Adding *K. ohmeri* and *L. lactis* increased the content of 2-methylbutanal and 3-methylbutanal in low-temperature moromi and raised their OAV (group K: 8.57 and 49.49; group L: 9.53 and 48.02). Conversely, adding *S. lloydii* decreased 2-methylbutanal and 3-methylbutanal content, possibly due to high aldehyde dehydrogenase or reductase activity converting them into 3-methylbutanoic acid and 3-methyl-1-butanol, thereby reducing their levels [39].

Yu et al. [40] showed that ethyl 3-methylbutyrate correlates positively with ethanol content. Ethyl 3-methylbutyrate content in group LT was much lower than in group MT but approached group MT levels in groups S and K, mirroring ethanol content trends. The OAV of ethyl acetate was higher in groups K (19.38), L (9.17), and S (22.69) than in group LT (8.11) and even exceeded that in group MT (20.89) for group S. Ethyl acetate forms via esterification of acetic acid and ethanol, with high-temperature fermentation favoring its formation [14]. The higher ethyl acetate content in group S than in group MT indicates that adding *S. lloydii* effectively promotes ethyl acetate formation, even surpassing high-temperature fermentation (group MT). Conversely, the OAV of 4-hydroxy-2(or 5)-ethyl-5(or 2)-methyl-3(2H)-furanone (HEMF, associated with roasted and burnt notes) was higher in group MT than in others, indicating that 30°C fermentation is more conducive to HEMF formation [14]. HMF, a precursor to HEMF, forms via the Pentose-Phosphate cycle and the Maillard reaction. It then reacts with acetaldehyde in soy sauce, catalyzed by yeast or Knoevenagel condensation, to form EDHMF, which is reduced to HEMF by yeast [14]. The higher HEMF content in group MT likely stems from the medium temperature (30°C), enhancing the Maillard reaction and HEMF formation.

## Conclusion

In conclusion, this study establishes a laboratory-driven Lingnan region seasonal temperature simulation system that efficiently replicates natural fermentation conditions while overcoming the temporal and resource constraints of field-based research. By precisely controlling temperature parameters (15–37°C), the system successfully identified *Staphylococcus lloydii*, *Leuconostoc lactis*, and *Kodamaea ohmeri* as pivotal strains whose low abundance under winter conditions (15°C) directly correlates with flavor deficiencies. The integration of targeted strain supplementation into low-temperature fermentation demonstrated significant flavor enhancement. Inoculating *S. lloydii* and *K. ohmeri* at critical fermentation stages elevated key aroma compounds (e.g., benzene, acetaldehyde, ethyl acetate, and 1-octen-3-ol) to levels comparable or superior to medium-temperature (30°C) fermentation. Notably, this approach required only 90 days of laboratory-scale trials, contrasting sharply with multi-year natural fermentation studies. This temperature-controlled simulation system offers the soy sauce industry a novel tool to efficiently and cost-effectively test the reinoculation of functional microbial strains, thereby ensuring consistent flavor profiles throughout the year.

## Supporting information

S1 File The minimal data set. (DOCX)

S2 File Graphical abstract. Schematic diagram of the laboratory-scale simulated fermentation system, sample collection, and experimental design. (a) 60 moromi samples were separately collected from three batches of LT, MT, HT, and NT on the 5th, 15th, 25th, 35th, and 60th day of fermentation. Schematic of the backfilling experimental design with collected sample and methodology types; (b) 90 moromi samples were collected from three batches of LT, MT, HT and NT at 5th, 15th, 25th, 35th, 60th and 90th day of fermentation (HT: 37°C; MT: 30°C; NT: 25°C; LT: 15°C; S: inoculated *S. lloydii*, 15°C; L: inoculated *L. lactis*, 15°C; S: inoculated *K. ohmeri*, 15°C). Points of different shapes represent different analysis methods used.
(TIF)

## References

[1] Zhao G, Liu C, Li S, Wang X, Yao Y. Exploring the flavor formation mechanism under osmotic conditions during soy sauce fermentation in Aspergillus oryzae by proteomic analysis. Food Funct. 2020;11(1):640–8. doi: 10.1039/c9fo02314c 31895399

[2] Syifaa AS, Jinap S, Sanny M, Khatib A. Chemical Profiling of Different Types of Soy Sauce and the Relationship with its Sensory Attributes. J Food Qual. 2016;39(6):714–25. doi: 10.1111/jfq.12240

[3] Qi Q, Huang J, Zhou R, Yang M, Zhang L, Peng C, et al. Characterizing microbial community and metabolites of Cantonese soy sauce. Food Biosci. 2021;40:100872. doi: 10.1016/j.fbio.2020.100872 34865765

[4] Zhou K, Siroli L, Patrignani F, Sun Y, Lanciotti R, Xu Z. Formation of Ethyl Carbamate during the Production Process of Cantonese Soy Sauce. Molecules. 2019;24(8):1474. doi: 10.3390/molecules24081474 30991675 PMC6514843

[5] Wen L, Lei J, Yang L, Kan Q, Wang P, Li J, et al. Metagenomics and untargeted metabolomics analyses to unravel the formation mechanism of characteristic metabolites in Cantonese soy sauce during different fermentation stages. Food Res Int. 2024;181:114116. doi: 10.1016/j.foodres.2024.114116 38448100

[6] Shi Y, Li Y, Yang K, Wei G, Huang A. A novel milk-derived peptide effectively inhibits Staphylococcus aureus: Interferes with cell wall synthesis, peptidoglycan biosynthesis disruption reaction mechanism, and its application in real milk system. Food Control. 2023;144:109374. doi: 10.1016/j.foodcont.2022.109374

[7] Zhuang M, Lin L, Zhao M, Dong Y, Sun-Waterhouse D, Chen H, et al. Sequence, taste and umami-enhancing effect of the peptides separated from soy sauce. Food Chem. 2016;206:174–81. doi: 10.1016/j.foodchem.2016.03.058 27041313

[8] Devanthi PVP, Gkatzionis K. Soy sauce fermentation: Microorganisms, aroma formation, and process modification. Food Res Int. 2019;120:364–74. doi: 10.1016/j.foodres.2019.03.010 31000250

[9] Zhang L, Xiong S, Du T, Xu Y, Madjirebaye P, Huang G, et al. Effect of microbiota succession on the dynamics of characteristic flavors and physicochemical properties during the soy sauce fermentation. Food Biosci. 2023;54:102883. doi: 10.1016/j.fbio.2023.102883

[10] Kilau FM, Mohamad NI, Saari N, Sani NA. Isolation, characterization and identification of lactic acid bacteria from fermented soy sauce. In: AIP Conf Proc. 2019. p. 2111. doi: 10.1063/1.5111245

[11] Wang J, Yan J, Zhang W, Zhang Y, Dong Z, Luo H, et al. Comparison of potential Wickerhamomyces anomalus to improve the quality of Cabernet Sauvignon wines by mixed fermentation with Saccharomyces cerevisiae. LWT Food Sci Technol. 2023;173:114285. doi: 10.1016/j.lwt.2022.114285

[12] Wang J, Zhao M, Xie N, Huang M, Feng Y. Community structure of yeast in fermented soy sauce and screening of functional yeast with potential to enhance the soy sauce flavor. Int J Food Microbiol. 2022;370:109652. doi: 10.1016/j.ijfoodmicro.2022.109652 35390573

[13] Feng Y, Su G, Zhao H, Cai Y, Cui C, Sun-Waterhouse D, et al. Characterisation of aroma profiles of commercial soy sauce by odour activity value and omission test. Food Chem. 2015;167:220–8. doi: 10.1016/j.foodchem.2014.06.057 25148982

[14] Feng YZ, Xie ZM, Huang MT, Tong X, Hou S, Ton H, et al. Decoding the molecular basis for temperature control by metabolomics to improve the taste quality of soy sauce fermented in winter. Food Bioscience. 2023;54:102889. doi: 10.1016/j.fbio.2023.102889

[15] Li X, Xu X, Wu C, Tong X, Ou S. Effect of Sequential Inoculation of Tetragenococcus halophilus and Wickerhamomyces anomalus on the Flavour Formation of Early-Stage Moromi Fermented at a Lower Temperature. Foods. 2023;12(18):3509. doi: 10.3390/foods12183509 37761218 PMC10530138

[16] Cui C, Zhao M, Li D, Zhao H, Sun W. Biochemical changes of traditional Chinese-type soy sauce produced in four seasons during processing. CyTA J Food. 2013;12(2):166–75. doi: 10.1080/19476337.2013.810673

[17] Ma J, Zhang J, Zhang L, Nie Y, Xu Y. Systematic analysis of key fermentation parameters influencing biogenic amines production in spontaneous fermentation of soy sauce. Food Biosci. 2023;52:102484. doi: 10.1016/j.fbio.2023.102484

[18] Feng Y, Zeng J, Lei H, Zhao M. Effect of fermentation containers on the taste characteristics and microbiota succession of soy sauce. Food Chem. 2024;448:139066. doi: 10.1016/j.foodchem.2024.139066 38569402

[19] Li X, Lee P, Taniasuri F, Liu S. Effects of yeast fermentation on transforming the volatile compounds of unsalted pork hydrolysate. Int J of Food Sci Tech. 2020;56(5):2291–303. doi: 10.1111/ijfs.14850

[20] Yu X, Lu Q, Wu C, Wang A, Huang G. Effect of ambient temperature on the quality of Cantonese-style high-salt liquid-state base soy sauce. Food Sci. 2023;44(22):55–63. doi: 10.7506/spkx1002-6630-20230118-141

[21] Lu Z-M, Wang Z-M, Zhang X-J, Mao J, Shi J-S, Xu Z-H. Microbial ecology of cereal vinegar fermentation: insights for driving the ecosystem function. Curr Opin Biotechnol. 2018;49:88–93. doi: 10.1016/j.copbio.2017.07.006 28843369

[22] Liu B, Li Y, Cao Z, Wang C. Effect of Tetragenococcus halophilus, Zygosaccharomyces rouxii, and Torulopsis versatilis addition sequence on soy sauce fermentation. Innov Food Sci Emerg Technol. 2021;69:102662. doi: 10.1016/j.ifset.2021.102662

[23] Gao P, Xia W, Li X, Liu S. Use of Wine and Dairy Yeasts as Single Starter Cultures for Flavor Compound Modification in Fish Sauce Fermentation. Front Microbiol. 2019;10:2300. doi: 10.3389/fmicb.2019.02300 31649641 PMC6794352

[24] Liang R, Huang J, Wu X, Xu Y, Fan J, Wu C, et al. Characterizing the metabolites and the microbial communities of the soy sauce mash affected by temperature and hydrostatic pressure. Food Res Int. 2019;123:801–8. doi: 10.1016/j.foodres.2019.06.002 31285030

[25] Nguyen NTH, Huang MB, Liu FY, Huang W-L, Tran H-T, Hsu T-W, et al. Deciphering microbial community dynamics along the fermentation course of soy sauce under different temperatures using metagenomic analysis. Biosci Microbiota Food Health. 2023;42(2):104–13. doi: 10.12938/bmfh.2022-012 37016686 PMC10067331

[26] Tan G, Wang Y, Hu M, Li X, Li X, Pan Z, et al. Comparative evaluation of the microbial diversity and metabolite profiles of Japanese-style and Cantonese-style soy sauce fermentation. Front Microbiol. 2022;13:976206. doi: 10.3389/fmicb.2022.976206 36003925 PMC9393507

[27] Fang F, Zhang J, Zhou J, Zhou Z, Li T, Lu L, et al. Accumulation of Citrulline by Microbial Arginine Metabolism during Alcoholic Fermentation of Soy Sauce. J Agric Food Chem. 2018;66(9):2108–13. doi: 10.1021/acs.jafc.7b06053 29457725

[28] He B, Li H, Hu Z, Zhang Y, Sun M, Qiu S, et al. Difference in microbial community and taste compounds between Mucor-type and Aspergillus-type Douchi during koji-making. Food Res Int. 2019;121:136–43. doi: 10.1016/j.foodres.2019.03.031 31108734

[29] Jia Y, Niu C-T, Zheng F-Y, Liu C-F, Wang J-J, Lu Z-M, et al. Development of a defined autochthonous starter through dissecting the seasonal microbiome of broad bean paste. Food Chem. 2021;357:129625. doi: 10.1016/j.foodchem.2021.129625 33864999

[30] Karra-Châabouni M, Ghamgui H, Bezzine S, Rekik A, Gargouri Y. Production of flavour esters by immobilized Staphylococcus simulans lipase in a solvent-free system. Process Biochem. 2006;41(7):1692–8. doi: 10.1016/j.procbio.2006.02.022

[31] Wang J, Wang Z, He F, Pan Z, Du Y, Chen Z, et al. Effect of microbial communities on flavor profile of Hakka rice wine throughout production. Food Chem X. 2024;21:101121. doi: 10.1016/j.fochx.2024.101121 38292683 PMC10824689

[32] Oro L, Feliziani E, Ciani M, Romanazzi G, Comitini F. Volatile organic compounds from Wickerhamomyces anomalus, Metschnikowia pulcherrima and Saccharomyces cerevisiae inhibit growth of decay causing fungi and control postharvest diseases of strawberries. Int J Food Microbiol. 2018;265:18–22. doi: 10.1016/j.ijfoodmicro.2017.10.027 29107842

[33] Gobbi M, Comitini F, Domizio P, Romani C, Lencioni L, Mannazzu I, et al. Lachancea thermotolerans and Saccharomyces cerevisiae in simultaneous and sequential co-fermentation: a strategy to enhance acidity and improve the overall quality of wine. Food Microbiol. 2013;33(2):271–81. doi: 10.1016/j.fm.2012.10.004 23200661

[34] Sharma A, Gupta G, Ahmad T, Kaur B, Hakeem KR. Tailoring cellular metabolism in lactic acid bacteria through metabolic engineering. J Microbiol Methods. 2020;170:105862. doi: 10.1016/j.mimet.2020.105862 32032637

[35] Xu Y, Wu M, Zhao D, Zheng J, Dai M, Li X, et al. Simulated Fermentation of Strong-Flavor Baijiu through Functional Microbial Combination to Realize the Stable Synthesis of Important Flavor Chemicals. Foods. 2023;12(3):644. doi: 10.3390/foods12030644 36766173 PMC9913964

[36] Zhao H, Xu J, Wang R, Liu X, Peng X, Guo S. Succession and Diversity of Microbial Flora during the Fermentation of Douchi and Their Effects on the Formation of Characteristic Aroma. Foods. 2023;12(2):329. doi: 10.3390/foods12020329 36673421 PMC9857697

[37] Guo Q, Peng J, Zhao J, Lei J, Huang Y, Shao B. Effects of Salinity on Physicochemical Properties, Flavor Compounds, and Bacterial Communities in Broad Bean Paste-Meju Fermentation. Foods. 2024;13(13):2108. doi: 10.3390/foods13132108 38998614 PMC11241834

[38] Zhao S, Tang F, Zhang Y, Cai W, Zhang Q, Zhao X, et al. Study on changes of bioactive substances and flavour characteristics during fermentation of jujube vinegar. Int J Food Sci Technol. 2024;59(12):9009–19. doi: 10.1111/ijfs.17415

[39] Liu M, Feng Y, Zhao M, Huang M. Decoding the molecular basis for temperature control by metabolomics to improve the taste quality of soy sauce fermented in winter. Food Biosci. 2023;54:102889. doi: 10.1016/j.fbio.2023.102889

[40] Yu H, Guo W, Xie T, Ai L, Tian H, Chen C. Aroma characteristics of traditional Huangjiu produced around Winter Solstice revealed by sensory evaluation, gas chromatography-mass spectrometry and gas chromatography-ion mobility spectrometry. Food Res Int. 2021;145:110421. doi: 10.1016/j.foodres.2021.110421 34112423

