# Supplementary material for: Simulate the natural four-season fermentation system for high-salt diluted-state soy sauce brewing: Application in flavor promotion regulation
Source: PLoS One. 2025 Oct 16;20(10):e0334707. doi: 10.1371/journal.pone.0334707 (PMC12530599; doi:10.1371/journal.pone.0334707)
Supplement: S1 File — (DOCX) [file pone.0334707.s001.docx]

**The minimal date set**

**Table 1 and 2 are the raw data of Figure 1:**

**Table 1. Raw data of relative abundance of the bacterial community in the moromi fermentation at different temperatures (HT: 37°C, MT: 30°C, NT: 25°C, LT: 15°C).**

|  | ***Weissella*** | ***Bacteroides*** | ***Staphylococcus*** | ***Leuconostoc*** | ***Lactococcus*** | ***Ralstonia*** | ***Lactobacillus*** | ***Enterococcus*** | ***Pseudomonas*** | ***Streptococcus*** | **Others** |
| --- | --- | --- | --- | --- | --- | --- | --- | --- | --- | --- | --- |
| **HT** | 56.96% | 2.29% | 11.37% | 2.18% | 1.26% | 0.80% | 0.87% | 1.96% | 2.89% | 1.01% | 18.40% |
| **MT** | 62.56% | 0.57% | 12.51% | 3.00% | 1.83% | 1.44% | 1.28% | 0.80% | 2.28% | 0.73% | 13.00% |
| **LT** | 72.50% | 0.73% | 6.34% | 1.28% | 0.86% | 2.66% | 1.32% | 0.33% | 0.61% | 0.11% | 13.25% |
| **NT** | 61.28% | 1.79% | 11.40% | 0.98% | 0.60% | 1.30% | 4.54% | 0.54% | 1.05% | 0.16% | 16.36% |

**Table 2. Relative abundance of the fungal community in the moromi fermentation at different temperatures (HT: 37°C, MT: 30°C, NT: 25°C, LT: 15°C).**

|  | **Aspergillus** | **Candida** | **Kodamaea** | **Gibberella** | **Aureobasidium** | **Millerozyma** | **Diutina** | **Issatchenkia** |
| --- | --- | --- | --- | --- | --- | --- | --- | --- |
| **HT** | 86.86% | 0.79% | 0.88% | 0.14% | 1.25% | 0.06% | 0.14% | 0.29% |
| **MT** | 70.84% | 0.79% | 21.27% | 0.06% | 0.05% | 0.04% | 0.81% | 1.19% |
| **LT** | 78.47% | 12.96% | 1.06% | 1.75% | 0.05% | 0.08% | 0.76% | 1.33% |
| **NT** | 63.49% | 12.05% | 12.81% | 0.68% | 0.03% | 2.49% | 1.26% | 1.53% |

**Table 3 and 4 are the raw data of Figure 2:**

**Table 3. Raw data of relative abundance of the bacterial community during the moromi fermentation at different temperatures (HT: 37°C, MT: 30°C, NT: 25°C, LT: 15°C).**

|  | ***Weissella*** | ***Bacteroides*** | ***Staphylococcus*** | ***Leuconostoc*** | ***Lactococcus*** | ***Ralstonia*** | ***Lactobacillus*** | ***Enterococcus*** | ***Pseudomonas*** | ***Streptococcus*** | **Others** |
| --- | --- | --- | --- | --- | --- | --- | --- | --- | --- | --- | --- |
| **HT5D** | 71.91% | 0.33% | 7.74% | 1.17% | 0.76% | 0.13% | 0.92% | 0.41% | 2.98% | 0.73% | 12.94% |
| **HT15D** | 67.32% | 0.01% | 10.89% | 3.40% | 3.47% | 0.39% | 0.54% | 1.37% | 0.59% | 1.90% | 10.11% |
| **HT25D** | 57.62% | 0.04% | 8.51% | 2.35% | 0.87% | 3.26% | 0.72% | 6.69% | 3.42% | 0.28% | 16.25% |
| **HT35D** | 53.18% | 0.11% | 18.45% | 1.98% | 0.48% | 0.02% | 0.72% | 0.80% | 3.87% | 0.94% | 19.44% |
| **HT60D** | 34.82% | 10.98% | 11.25% | 1.97% | 0.73% | 0.19% | 1.48% | 0.52% | 3.60% | 1.22% | 33.24% |
| **MT5D** | 72.10% | 2.68% | 6.27% | 0.71% | 0.45% | 0.09% | 2.55% | 0.13% | 0.97% | 0.09% | 13.97% |
| **MT15D** | 68.85% | 0.01% | 17.60% | 2.11% | 0.79% | 0.34% | 0.57% | 1.41% | 1.17% | 0.29% | 6.86% |
| **MT25D** | 68.84% | 0.03% | 10.57% | 1.46% | 0.32% | 4.55% | 0.43% | 1.35% | 4.86% | 0.17% | 7.41% |
| **MT35D** | 55.98% | 0.08% | 17.96% | 2.23% | 0.72% | 0.06% | 1.05% | 0.47% | 1.98% | 1.29% | 18.17% |
| **MT60D** | 47.04% | 0.06% | 10.14% | 8.49% | 6.90% | 2.12% | 1.80% | 0.67% | 2.38% | 1.84% | 18.56% |
| **LT5D** | 83.02% | 0.39% | 4.35% | 0.54% | 0.51% | 0.06% | 2.61% | 0.16% | 0.40% | 0.08% | 7.88% |
| **LT15D** | 72.14% | 0.07% | 5.07% | 2.23% | 2.08% | 4.37% | 0.26% | 0.41% | 0.26% | 0.20% | 12.91% |
| **LT25D** | 81.48% | 0.18% | 1.97% | 1.24% | 0.25% | 8.77% | 0.21% | 0.32% | 0.69% | 0.05% | 4.85% |
| **LT35D** | 68.26% | 0.04% | 11.03% | 1.95% | 1.09% | 0.06% | 1.60% | 0.25% | 0.94% | 0.13% | 14.65% |
| **LT60D** | 57.60% | 2.97% | 9.30% | 0.47% | 0.38% | 0.04% | 1.94% | 0.53% | 0.77% | 0.06% | 25.93% |
| **NT5D** | 81.44% | 2.15% | 3.60% | 0.54% | 0.18% | 0.12% | 3.24% | 0.11% | 0.26% | 0.09% | 8.26% |
| **NT15D** | 74.98% | 0.11% | 10.90% | 1.62% | 1.03% | 0.57% | 2.47% | 0.47% | 1.47% | 0.31% | 6.07% |
| **NT25D** | 54.74% | 0.67% | 13.36% | 0.89% | 0.48% | 5.40% | 3.10% | 0.81% | 1.57% | 0.21% | 18.77% |
| **NT35D** | 52.46% | 0.54% | 14.66% | 1.39% | 1.12% | 0.10% | 1.07% | 0.31% | 1.19% | 0.14% | 27.02% |
| **NT60D** | 42.77% | 5.49% | 14.48% | 0.44% | 0.20% | 0.30% | 12.83% | 1.00% | 0.77% | 0.04% | 21.69% |

**Table 4. Raw data of relative abundance of the fungal community during the moromi fermentation at different temperatures (HT: 37°C, MT: 30°C, NT: 25°C, LT: 15°C).**

|  | ***Aspergillus*** | ***Candida*** | ***Kodamaea*** | ***Gibberella*** | ***Aureobasidium*** | ***Millerozyma*** | ***Diutina*** | ***Issatchenkia*** | **Others** |
| --- | --- | --- | --- | --- | --- | --- | --- | --- | --- |
| **HT5D** | 86.93% | 2.59% | 1.51% | 0.01% | 0.00% | 0.15% | 0.45% | 0.75% | 7.60% |
| **HT15D** | 74.48% | 0.34% | 1.24% | 0.51% | 0.08% | 0.11% | 0.11% | 0.26% | 22.86% |
| **HT25D** | 87.22% | 0.65% | 0.39% | 0.16% | 0.01% | 0.03% | 0.10% | 0.23% | 11.20% |
| **HT35D** | 88.76% | 0.16% | 0.69% | 0.00% | 6.08% | 0.00% | 0.02% | 0.11% | 4.17% |
| **HT60D** | 96.90% | 0.20% | 0.55% | 0.00% | 0.06% | 0.00% | 0.02% | 0.11% | 2.16% |
| **MT5D** | 83.30% | 0.89% | 0.39% | 0.25% | 0.00% | 0.12% | 1.84% | 1.27% | 11.95% |
| **MT15D** | 87.67% | 1.54% | 5.37% | 0.01% | 0.00% | 0.01% | 1.88% | 2.43% | 1.08% |
| **MT25D** | 72.96% | 0.81% | 18.40% | 0.02% | 0.01% | 0.06% | 0.20% | 0.59% | 6.96% |
| **MT35D** | 63.04% | 0.42% | 32.63% | 0.00% | 0.13% | 0.00% | 0.10% | 1.47% | 2.21% |
| **MT60D** | 47.22% | 0.28% | 49.55% | 0.00% | 0.11% | 0.02% | 0.06% | 0.20% | 2.56% |
| **LT5D** | 99.76% | 0.10% | 0.01% | 0.00% | 0.00% | 0.00% | 0.02% | 0.04% | 0.06% |
| **LT15D** | 66.58% | 29.01% | 0.39% | 0.01% | 0.00% | 0.02% | 1.56% | 1.05% | 1.38% |
| **LT25D** | 78.97% | 13.19% | 1.37% | 0.02% | 0.00% | 0.20% | 0.92% | 2.36% | 2.98% |
| **LT35D** | 68.79% | 10.16% | 1.82% | 8.48% | 0.12% | 0.06% | 0.53% | 1.66% | 8.39% |
| **LT60D** | 78.25% | 12.35% | 1.68% | 0.24% | 0.12% | 0.14% | 0.76% | 1.55% | 4.92% |
| **NT5D** | 57.68% | 22.51% | 0.23% | 1.62% | 0.00% | 0.02% | 3.44% | 3.70% | 10.80% |
| **NT15D** | 66.07% | 27.42% | 1.59% | 0.00% | 0.00% | 0.15% | 1.55% | 1.73% | 1.47% |
| **NT25D** | 86.02% | 5.83% | 1.77% | 1.35% | 0.04% | 0.13% | 0.72% | 1.06% | 3.08% |
| **NT35D** | 84.17% | 3.97% | 4.58% | 0.18% | 0.11% | 0.43% | 0.43% | 0.92% | 5.21% |
| **NT60D** | 23.55% | 0.51% | 55.86% | 0.26% | 0.01% | 11.72% | 0.18% | 0.27% | 7.65% |

**Table 5 and 6 are the raw data of Figure 3:**

**Table 5. Raw data of LEfSe analysis of microbial community of moromi at different temperatures (HT: 37°C, MT: 30°C, NT: 25°C, LT: 15°C).**

| **Microbe** | **Group** | **LAD (log10)** |
| --- | --- | --- |
| **Bacterial** |  |  |
| *Staphylococcus lentus* | NT | 3.82 |
| *Lactobacillus intestinalis* | NT | 3.75 |
| *Staphylococcus equorum* | NT | 3.51 |
| *Staphylococcales* | MT | 4.50 |
| *Staphylococcus* | MT | 4.50 |
| *Staphylococcaceae* | MT | 4.50 |
| *Pseudomonadales* | HT | 4.09 |
| *Actinobacteria* | HT | 3.82 |
| **Fungal** |  |  |
| *Saccharomycetes* | NT | 5.12 |
| *Saccharomycetales* | NT | 5.12 |
| *Debaryomycetacea* | NT | 4.12 |
| *Millerozyma* | NT | 4.12 |
| *Millerozyma farinosa* | NT | 4.12 |
| *Issatchenkia orientalis* | NT | 3.79 |
| *Issatchenkia* | NT | 3.79 |
| *Saccharomycetaceae* | NT | 3.79 |
| *Diutina rugosa* | NT | 3.79 |
| *Diutina* | NT | 3.79 |
| *Saccharomycetes_fam_Incetae_Sedis* | LT | 4.68 |
| *Vishniacozyma victoriae* | HT | 3.83 |
| *Vishniacozyma* | HT | 3.79 |

**Table 6. Raw data of simper analysis of the microbial community differences between group MT and group LT during moromi fermentation (MT: 30°C, LT: 15°C).**

| **Microbe** | **Differential contribution** |
| --- | --- |
| **Bacterial** |  |
| *Weissella* | 0.32 |
| *Staphylococcus* | 0.15 |
| *Ralstonia* | 0.07 |
| *Leuconostoc* | 0.05 |
| *Pseudomonas* | 0.04 |
| *Lactococcus* | 0.03 |
| *Lactobacillus* | 0.02 |
| *Bacteroides* | 0.01 |
| *Enterococcus* | 0.01 |
| **Fungal** |  |
| *Aspergillus* | 0.39 |
| *Kodamaea* | 0.31 |
| *Candida* | 0.17 |
| *Gibberella* | 0.03 |
| *Isstachenkia* | 0.02 |
| *Diutina* | 0.02 |
| *Millerozyme* | 0.01 |
| *Aureobasidium* | 0.01 |

**Table 7 and 8 are the raw data of Figure 4:**

**Table 7. Row data of spearman correlation analysis of the relationship between dominant bacterial and physicochemical factors free amino acid as well as flavor indexes of moromi.**

| **Microbe**  **Indexes** | **Spearman’s rank coefficient of correlation** | | | | | | | | | |
| --- | --- | --- | --- | --- | --- | --- | --- | --- | --- | --- |
|  | *Weissella* | *Bacteroides* | *Staphylococcus* | *Leuconostoc* | *Lactococcus* | *Ralstonia* | *Lactobacillus* | *Enterococcus* | *Pseudomonas* | *Streptococcus* |
| Asp | -0.148 | -0.309 | 0.241 | 0.930 | 0.937 | -0.150 | -0.881 | 0.462 | 0.700 | 0.759 |
| Glu | 0.971 | -0.872 | -0.723 | -0.183 | -0.081 | 0.966 | -0.124 | -0.826 | -0.734 | -0.671 |
| Thr | 0.579 | -0.631 | -0.523 | 0.421 | 0.485 | 0.581 | -0.866 | -0.058 | 0.024 | 0.138 |
| Ser | 0.016 | -0.401 | 0.071 | 0.863 | 0.884 | 0.015 | -0.931 | 0.368 | 0.581 | 0.657 |
| Gly | -0.654 | -0.033 | 0.927 | 0.767 | 0.735 | -0.667 | -0.015 | 0.308 | 0.675 | 0.606 |
| Ala | -0.700 | 0.893 | 0.257 | 0.045 | -0.046 | -0.687 | -0.218 | 0.918 | 0.647 | 0.65 |
| Lys | 0.713 | -0.825 | -0.300 | -0.188 | -0.101 | 0.701 | 0.338 | -0.964 | -0.740 | -0.751 |
| Pro | -0.765 | 0.222 | 0.801 | 0.871 | 0.816 | -0.769 | -0.431 | 0.746 | 0.966 | 0.945 |
| Val | -0.253 | -0.179 | 0.632 | 0.089 | 0.089 | -0.268 | 0.678 | -0.388 | -0.086 | -0.194 |
| Met | 0.634 | -0.689 | -0.249 | -0.329 | -0.253 | 0.622 | 0.525 | -0.975 | -0.792 | -0.822 |
| Ile | -0.539 | -0.162 | 0.793 | 0.935 | 0.915 | -0.55 | -0.338 | 0.387 | 0.764 | 0.733 |
| Leu | -0.477 | -0.19 | 0.689 | 0.984 | 0.969 | -0.485 | -0.511 | 0.451 | 0.802 | 0.795 |
| Tyr | -0.696 | 0.347 | 0.567 | 0.783 | 0.724 | -0.694 | -0.634 | 0.901 | 0.984 | 0.999 |
| Phe | -0.642 | 0.114 | 0.674 | 0.925 | 0.882 | -0.646 | -0.596 | 0.735 | 0.956 | 0.958 |
| His | 0.759 | -0.595 | -0.493 | -0.560 | -0.483 | 0.752 | 0.528 | -0.990 | -0.939 | -0.950 |
| Arg | 0.513 | -0.535 | -0.162 | -0.427 | -0.366 | 0.502 | 0.683 | -0.938 | -0.789 | -0.839 |
| Cys | 0.689 | -0.166 | -0.709 | -0.904 | -0.856 | 0.692 | 0.554 | -0.758 | -0.969 | -0.965 |
| RS | -0.944 | 0.904 | 0.697 | 0.038 | -0.064 | -0.940 | 0.281 | 0.732 | 0.613 | 0.539 |
| pH | 0.896 | -0.566 | -0.745 | -0.641 | -0.559 | 0.894 | 0.342 | -0.926 | -0.979 | -0.955 |
| TA | -0.901 | 0.545 | 0.770 | 0.658 | 0.578 | -0.900 | -0.328 | 0.909 | 0.980 | 0.953 |
| TN | -0.922 | 0.448 | 0.907 | 0.690 | 0.614 | -0.925 | -0.169 | 0.772 | 0.934 | 0.881 |
| Pyrazines | -0.712 | 0.585 | 0.426 | 0.540 | 0.465 | -0.705 | -0.570 | 0.988 | 0.917 | 0.937 |
| Sulfurs | -0.809 | 0.878 | 0.422 | 0.174 | 0.079 | -0.799 | -0.207 | 0.956 | 0.754 | 0.744 |
| Furans | -0.838 | 0.529 | 0.668 | 0.669 | 0.592 | -0.835 | -0.450 | 0.946 | 0.988 | 0.98 |
| Phenols | -0.542 | -0.121 | 0.89 | 0.596 | 0.576 | -0.557 | 0.227 | 0.060 | 0.445 | 0.357 |
| Ketones | -0.573 | 0.217 | 0.469 | 0.830 | 0.784 | -0.571 | -0.745 | 0.846 | 0.948 | 0.979 |
| Esters | 0.704 | -0.996 | -0.306 | 0.341 | 0.435 | 0.693 | -0.326 | -0.634 | -0.328 | -0.279 |
| Acids | -0.223 | 0.094 | 0.033 | 0.659 | 0.638 | -0.216 | -0.935 | 0.734 | 0.725 | 0.806 |
| Aldehydes | -0.857 | 0.518 | 0.711 | 0.683 | 0.606 | -0.855 | -0.416 | 0.929 | 0.991 | 0.976 |
| Alcohols | -0.012 | -0.611 | 0.512 | 0.496 | 0.533 | -0.030 | 0.185 | -0.414 | 0.032 | -0.025 |

**Table 8. Row data of spearman correlation analysis of the relationship between dominant fungal and physicochemical factors, free amino acid as well as flavor indexes of moromi.**

| **Microbe**  **Indexes** | **Spearman’s rank coefficient of correlation** | | | | | | | |
| --- | --- | --- | --- | --- | --- | --- | --- | --- |
|  | *Aspergillus* | *Candida* | *Kodamaea* | *Gibberella* | *Aureobasidium* | *Millerozyma* | *Diutina* | *Issatchenkia* |
| Asp | 0.543 | -0.836 | 0.152 | -0.462 | 0.348 | -0.883 | -0.666 | -0.562 |
| Glu | -0.200 | 0.549 | 0.030 | 0.767 | -0.745 | -0.229 | 0.388 | 0.651 |
| Thr | 0.562 | -0.189 | -0.239 | 0.326 | -0.004 | -0.913 | -0.468 | -0.187 |
| Ser | 0.583 | -0.731 | 0.064 | -0.301 | 0.287 | -0.944 | -0.661 | -0.509 |
| Gly | -0.282 | -0.753 | 0.808 | -0.908 | 0.046 | -0.013 | 0.012 | -0.145 |
| Ala | 0.663 | -0.436 | -0.557 | -0.415 | 0.967 | -0.101 | -0.735 | -0.878 |
| Lys | -0.717 | 0.555 | 0.507 | 0.485 | -0.991 | 0.223 | 0.809 | 0.937 |
| Pro | 0.288 | -0.984 | 0.361 | -0.935 | 0.551 | -0.383 | -0.54 | -0.661 |
| Val | -0.913 | 0.018 | 0.905 | -0.398 | -0.583 | 0.634 | 0.764 | 0.598 |
| Met | -0.821 | 0.647 | 0.506 | 0.476 | -0.995 | 0.420 | 0.911 | 0.989 |
| Ile | -0.027 | -0.880 | 0.688 | -0.857 | 0.143 | -0.340 | -0.224 | -0.301 |
| Leu | 0.145 | -0.925 | 0.563 | -0.804 | 0.231 | -0.511 | -0.376 | -0.411 |
| Tyr | 0.618 | -0.959 | -0.004 | -0.780 | 0.787 | -0.565 | -0.812 | -0.885 |
| Phe | 0.410 | -0.999 | 0.283 | -0.848 | 0.559 | -0.556 | -0.637 | -0.701 |
| His | -0.694 | 0.837 | 0.226 | 0.704 | -0.930 | 0.433 | 0.856 | 0.963 |
| Arg | -0.898 | 0.685 | 0.512 | 0.420 | -0.961 | 0.592 | 0.973 | 0.996 |
| Cys | -0.394 | 0.999 | -0.286 | 0.876 | -0.580 | 0.508 | 0.627 | 0.710 |
| RS | 0.085 | -0.412 | -0.025 | -0.698 | 0.663 | 0.384 | -0.256 | -0.537 |
| pH | -0.432 | 0.897 | -0.073 | 0.893 | -0.794 | 0.256 | 0.657 | 0.827 |
| TA | 0.399 | -0.905 | 0.116 | -0.911 | 0.767 | -0.245 | -0.631 | -0.804 |
| TN | 0.155 | -0.888 | 0.361 | -0.987 | 0.581 | -0.104 | -0.420 | -0.622 |
| Pyrazines | 0.746 | -0.814 | -0.290 | -0.650 | 0.946 | -0.475 | -0.891 | -0.981 |
| Sulfurs | 0.594 | -0.558 | -0.401 | -0.571 | 0.957 | -0.091 | -0.714 | -0.886 |
| Furans | 0.531 | -0.915 | 0.001 | -0.845 | 0.830 | -0.365 | -0.741 | -0.879 |
| Phenols | -0.544 | -0.534 | 0.927 | -0.790 | -0.202 | 0.209 | 0.297 | 0.130 |
| Ketones | 0.670 | -0.955 | -0.022 | -0.703 | 0.740 | -0.688 | -0.843 | -0.868 |
| Esters | -0.215 | 0.071 | 0.414 | 0.313 | -0.687 | -0.436 | 0.270 | 0.500 |
| Acids | 0.898 | -0.740 | -0.360 | -0.319 | 0.731 | -0.888 | -0.957 | -0.867 |
| Aldehydes | 0.481 | -0.922 | 0.057 | -0.876 | 0.799 | -0.334 | -0.701 | -0.849 |
| Alcohols | -0.690 | -0.227 | 0.977 | -0.378 | -0.638 | 0.107 | 0.542 | 0.515 |

**Table 9 and 10 are the raw data of Figure 5:**

**Table 9. Row data of physicochemical indexes in five groups of moromi during fermentation (MT: 30°C; LT: 15°C; *S. lloydii*: inoculated *S. lloydii*, 15°C; *L. lactis*: inoculated *L. lactis*, 15°C; *S. ohmeri*: inoculated *K. ohmeri*, 15°C).**

| **Fermentation time (d)** | | **Groups** | | | | | | | | | | | | | | | | | | | | | | | | | | | | | | | | | | |
| --- | --- | --- | --- | --- | --- | --- | --- | --- | --- | --- | --- | --- | --- | --- | --- | --- | --- | --- | --- | --- | --- | --- | --- | --- | --- | --- | --- | --- | --- | --- | --- | --- | --- | --- | --- | --- |
|  |  | **CK** | | | | | | | **MT** | | | | | | | ***K. ohmeri*** | | | | | | ***S. lloydii*** | | | | | | | ***L. lactis*** | | | | | | | |
| **Total acid （g/100 mL）** | | | | | | | | | | | | | | | | | | | | | | | | | | | | | | | | | | | | |
| 5 | | 1.144 | | 1.074 | | | 1.121 | | 1.417 | | 1.383 | | | 1.403 | | 1.243 | | 1.239 | | | 1.246 | 1.211 | | | 1.145 | | | 1.183 | 1.189 | | | 1.121 | | | 1.171 | |
| 15 | | 1.363 | | 1.308 | | | 1.344 | | 1.455 | | 1.392 | | | 1.423 | | 1.340 | | 1.389 | | | 1.365 | 1.270 | | | 1.265 | | | 1.271 | 1.275 | | | 1.187 | | | 1.239 | |
| 25 | | 1.395 | | 1.353 | | | 1.374 | | 1.499 | | 1.355 | | | 1.435 | | 1.368 | | 1.353 | | | 1.361 | 1.382 | | | 1.311 | | | 1.359 | 1.2943 | | | 1.373 | | | 1.332 | |
| 35 | | 1.434 | | 1.376 | | | 1.412 | | 1.518 | | 1.576 | | | 1.556 | | 1.437 | | 1.473 | | | 1.463 | 1.479 | | | 1.561 | | | 1.522 | 1.494 | | | 1.552 | | | 1.521 | |
| 60 | | 1.538 | | 1.466 | | | 1.509 | | 1.79 | | 1.748 | | | 1.774 | | 1.538 | | 1.620 | | | 1.583 | 1.502 | | | 1.57 | | | 1.549 | 1.592 | | | 1.550 | | | 1.572 | |
| 90 | | 1.608 | 1.544 | | | 1.586 | | | 1.8 | 1.860 | | | 1.836 | | | 1.530 | | 1.606 | | 1.574 | | 1.572 | | 1.592 | | | 1.577 | | 1.646 | | 1.576 | | | 1.620 | |  |
| **Amino acid nitrogen（g/100 mL）** | | | | | | | | | | | | | | | | | | | | | | | | | | | | | | | | | | | |  |
| 5 | | 0.664 | 0.652 | | | 0.662 | | 0.802 | | 0.794 | | | 0.813 | | | 0.69 | | 0.708 | | 0.702 | | 0.702 | | 0.686 | | | 0.693 | | 0.698 | | 0.657 | | | 0.688 | |  |
| 15 | | 0.844 | | 0.824 | 0.833 | | | | 0.914 | 0.880 | | 0.911 | | | 0.852 | | 0.883 | | 0.877 | | | 0.844 | 0.86 | | | 0.855 | | | 0.849 | 0.776 | | | 0.812 | | |  |
| 25 | | 0.960 | | 0.924 | 0.939 | | | | 0.944 | 0.964 | | 0.956 | | | 0.919 | | 0.898 | | 0.915 | | | 0.957 | 0.927 | | | 0.943 | | | 0.874 | 0.929 | | | 0.905 | | |  |
| 35 | | 1.000 | | 0.964 | 0.984 | | | | 0.980 | 1.028 | | 1.005 | | | 0.968 | | 1.026 | | 1.004 | | | 1.024 | 1.059 | | | 1.042 | | | 1.013 | 1.037 | | | 1.033 | | |  |
| 60 | | 1.092 | 1.046 | | | 1.077 | | 1.020 | | 1.018 | | | 1.024 | | | 1.050 | | 1.124 | | 1.093 | | 1.056 | | 1.120 | | | 1.096 | | 1.096 | | 1.084 | | | 1.095 | |  |
| 90 | | 1.148 | | 1.090 | | | 1.123 | | 1.102 | | 1.178 | | | 1.148 | | 1.084 | | 1.154 | | | 1.129 | 1.146 | | | 1.174 | | | 1.168 | 1.180 | | | 1.164 | | | 1.172 | |
| **Reducing sugar （g/100 mL）** | | | | | | | | | | | | | | | | | | | | | | | | | | | | | | | | | | | | |
| 5 | 4.528 | | 4.706 | | | 4.629 | | 5.771 | | 5.616 | | | 5.693 | | 4.791 | | 4.481 | | | 4.648 | 3.407 | | | 2.783 | | | 3.103 | 4.645 | | | 3.669 | | | 4.166 | |  |
| 15 | 5.072 | | 5.588 | | | 5.332 | | 5.959 | | 6.118 | | | 6.046 | | 5.250 | | 4.495 | | | 4.877 | 3.167 | | | 3.885 | | | 3.532 | 4.945 | | | 4.598 | | | 4.773 | |  |
| 25 | 5.410 | | 6.414 | | | 5.914 | | 6.287 | | 5.532 | | | 5.913 | | 6.006 | | 5.180 | | | 5.593 | 4.739 | | | 4.636 | | | 4.695 | 5.283 | | | 5.316 | | | 5.302 | |  |
| 35 | 5.682 | | 6.071 | | | 5.883 | | 5.865 | | 5.865 | | | 5.877 | | 5.790 | | 5.222 | | | 5.513 | 3.866 | | | 4.063 | | | 3.963 | 4.955 | | | 4.598 | | | 4.784 | |  |
| 60 | 4.959 | | 5.757 | | | 5.367 | | 3.927 | | 3.566 | | | 3.753 | | 5.142 | | 4.265 | | | 4.703 | 2.862 | | | 3.885 | | | 3.371 | 3.604 | | | 3.327 | | | 3.471 | |  |
| 90 | 4.368 | 4.622 | | | 4.502 | | 1.563 | | 1.408 | | | 1.498 | | | 3.167 | | 3.472 | | 3.321 | | 2.417 | | 2.806 | | | 2.619 | | 2.829 | | 2.890 | | | 2.865 | |  |  |

**Table 10. Means and standard deviations of physicochemical indexes in five groups of moromi during fermentation (MT: 30°C; LT: 15°C; *S. lloydii*: inoculated *S. lloydii*, 15°C; *L. lactis*: inoculated *L. lactis*, 15°C; *S. ohmeri*: inoculated *K. ohmeri*, 15°C).**

| **Fermentation time (d)** | **CK** | **MT** | ***K. ohmeri*** | ***S. lloydii*** | ***L. lactis*** |  |
| --- | --- | --- | --- | --- | --- | --- |
| **Total acid （g/100 mL）** |  |  |  |  |  |  |
| **5** | 1.11±0.04 | 1.40±0.02 | 1.24±0.00 | 1.18±0.03 | 1.16±0.04 |  |
| **15** | 1.34±0.03 | 1.42±0.03 | 1.36±0.02 | 1.27±0.00 | 1.23±0.04 |  |
| **25** | 1.37±0.02 | 1.43±0.07 | 1.36±0.01 | 1.35±0.04 | 1.33±0.04 |  |
| **35** | 1.41±0.03 | 1.55±0.03 | 1.46±0.02 | 1.52±0.04 | 1.52±0.03 |  |
| **60** | 1.50±0.04 | 1.77±0.02 | 1.58±0.04 | 1.54±0.03 | 1.57±0.02 |  |
| **90** | 1.58±0.03 | 1.83±0.03 | 1.57±0.04 | 1.58±0.01 | 1.61±0.04 |  |
| **Amino acid nitrogen**  **（g/100 mL）** | |  |  |  |  |  |
| **5** | | 0.66±0.01 | 0.80±0.01 | 0.70±0.01 | 0.69±0.01 | 0.68±0.02 |
| **15** | | 0.83±0.01 | 0.90±0.02 | 0.87±0.02 | 0.85±0.01 | 0.81±0.04 |
| **25** | | 0.94±0.02 | 0.95±0.01 | 0.91±0.01 | 0.94±0.02 | 0.90±0.03 |
| **35** | | 0.98±0.02 | 1.00±0.02 | 1.00±0.03 | 1.04±0.02 | 1.03±0.01 |
| **60** | | 1.07±0.02 | 1.02±0.00 | 1.09±0.04 | 1.09±0.03 | 1.09±0.01 |
| **90** | | 1.12±0.03 | 1.14±0.04 | 1.12±0.04 | 1.16±0.01 | 1.17±0.01 |
| **Reducing sugar （g/100 mL）** | |  |  |  |  |  |
| **5** | | 4.62±0.09 | 5.69±0.08 | 4.64±0.16 | 3.10±0.31 | 4.16±0.49 |
| **15** | | 5.33±0.26 | 6.04±0.08 | 4.87±0.38 | 3.53±0.36 | 4.77±0.17 |
| **25** | | 5.91±0.50 | 5.91±0.38 | 5.59±0.41 | 4.69±0.05 | 5.30±0.02 |
| **35** | | 5.88±0.19 | 5.87±0.01 | 5.51±0.28 | 3.96±0.10 | 4.78±0.18 |
| **60** | | 5.36±0.40 | 3.75±0.18 | 4.70±0.44 | 3.37±0.51 | 3.47±0.14 |
| **90** | | 4.50±0.13 | 1.49±0.08 | 3.32±0.15 | 2.61±0.19 | 2.86±0.03 |

**Table 11 and 12 are the raw data of Figure 6:**

**Table 12. Row data of volatile compounds of five groups of moromi during fermentation (MT: 30°C; LT: 15°C; *S. lloydii*: inoculated *S. lloydii*, 15°C; *L. lactis*: inoculated *L. lactis*, 15°C; *K. ohmeri*: inoculated *K. ohmeri*, 15°C).**

| **Compounds** | **Content of volatile compounds (μg/L)** | | | | | | | | | | | | | | |
| --- | --- | --- | --- | --- | --- | --- | --- | --- | --- | --- | --- | --- | --- | --- | --- |
|  | **LT** | | | ***K. ohmeri*** | | | ***L. lactis*** | | | ***S. lloydii*** | | | **MT** | | |
| **Alcohol (14 types)** |  |  |  |  |  |  |  |  |  |  |  |  |  |  |  |
| Methanethiol | 0.13 | 0.19 | 0.27 | 0.22 | 0.24 | 0.27 | 0.21 | 0.23 | 0.22 | 0.00 | 0.00 | 0.00 | 0.33 | 0.32 | 0.32 |
| 2-Naphthalenethiol | 0.32 | 0.39 | 0.28 | 0.66 | 0.59 | 0.55 | 0.00 | 0.00 | 0.00 | 0.00 | 0.00 | 0.00 | 0.39 | 0.33 | 0.44 |
| 1-Dodecanol | 0.00 | 0.00 | 0.00 | 1.03 | 1.01 | 0.84 | 3.77 | 3.15 | 3.48 | 1.23 | 1.11 | 1.01 | 0.00 | 0.00 | 0.00 |
| Ethanol | 369.68 | 380.96 | 333.06 | 1017.61 | 1001.76 | 996.09 | 476.06 | 499.41 | 504.08 | 1073.1 | 1006.25 | 1033.40 | 1107.39 | 1027.21 | 1126.63 |
| 1-Propanol | 3.20 | 3.50 | 3.02 | 6.14 | 4.35 | 5.72 | 5.72 | 4.27 | 6.88 | 6.48 | 4.54 | 6.04 | 20.14 | 15.08 | 18.22 |
| 1-Propanol, 2-methyl- | 9.22 | 9.31 | 8.93 | 18.29 | 19.02 | 15.83 | 9.01 | 9.12 | 8.88 | 20.28 | 20.46 | 17.42 | 11.27 | 11.51 | 11.73 |
| 1-Butanol | 0.19 | 0.25 | 0.39 | 2.11 | 2.03 | 1.94 | 0.52 | 0.55 | 0.49 | 4.00 | 4.02 | 4.09 | 5.33 | 5.30 | 5.22 |
| Propanal, 2-methyl- | 2.56 | 2.99 | 2.45 | 3.99 | 4.01 | 3.15 | 1.31 | 1.40 | 1.19 | 1.22 | 1.31 | 1.20 | 5.38 | 5.32 | 5.44 |
| 1-Octen-3-ol | 32.35 | 35.34 | 41.03 | 47.15 | 47.15 | 47.15 | 39.19 | 39.19 | 39.20 | 65.20 | 65.21 | 65.20 | 13.48 | 17.52 | 12.74 |
| 1-Hexanol, 2-ethyl- | 9.29 | 10.11 | 8.18 | 12.35 | 11.99 | 10.13 | 11.89 | 11.97 | 11.95 | 12.93 | 12.87 | 12.61 | 8.31 | 7.99 | 8.32 |
| Phenylethyl Alcohol | 7.69 | 5.72 | 10.13 | 13.94 | 18.03 | 16.19 | 13.63 | 8.18 | 15.59 | 28.75 | 31.16 | 18.07 | 74.78 | 56.80 | 65.44 |
| 3-Methyl-1-butanol | 35.00 | 48.16 | 38.29 | 66.55 | 48.78 | 56.44 | 41.83 | 30.24 | 27.98 | 95.44 | 52.78 | 76.29 | 97.48 | 83.25 | 68.77 |
| 1-Hexanol | 1.89 | 1.33 | 1.54 | 2.34 | 2.35 | 2.14 | 2.31 | 2.2 | 2.15 | 2.77 | 2.29 | 2.38 | 1.19 | 1.17 | 1.13 |
| Maltol | 2.71 | 2.66 | 2.80 | 7.42 | 5.88 | 7.68 | 6.09 | 3.15 | 6.46 | 5.97 | 3.13 | 5.48 | 10.26 | 7.42 | 10.91 |
| **Total** | 474.32 | 500.68 | 450.5 | 1199.8 | 1167.19 | 1164.12 | 611.54 | 613.06 | 628.55 | 1317.37 | 1205.13 | 1243.19 | 1355.73 | 1239.22 | 1335.31 |
| **Esters (23 types)** |  |  |  |  |  |  |  |  |  |  |  |  |  |  |  |
| Ethyl propionate | 0.28 | 0.26 | 0.28 | 0.61 | 0.54 | 0.66 | 0.33 | 0.35 | 0.38 | 0.99 | 0.83 | 0.65 | 1.22 | 1.32 | 1.14 |
| Methyl butyrate | 1.38 | 1.29 | 1.50 | 0.65 | 0.66 | 0.69 | 1.12 | 1.13 | 1.27 | 0.44 | 0.41 | 0.47 | 1.21 | 1.22 | 1.02 |
| Ethyl 3-methylbutyrate | 1.35 | 1.14 | 2.29 | 1.09 | 1.08 | 1.09 | 1.99 | 1.88 | 1.96 | 0.94 | 0.76 | 0.91 | 0.55 | 0.59 | 0.50 |
| Methyl hexanoate | 1.28 | 0.99 | 1.14 | 1.19 | 1.33 | 1.01 | 1.44 | 1.43 | 1.42 | 1.22 | 1.32 | 1.15 | 0.88 | 0.84 | 0.85 |
| Ethyl hexanoate | 2.67 | 2.11 | 1.87 | 1.82 | 1.88 | 1.83 | 0.77 | 0.79 | 0.77 | 2.56 | 2.33 | 2.65 | 0.44 | 0.43 | 0.45 |
| Ethyl phenylacetate | 0.40 | 0.43 | 0.41 | 1.30 | 1.79 | 2.32 | 0.72 | 0.74 | 0.74 | 3.11 | 1.64 | 2.38 | 3.99 | 4.50 | 3.38 |
| Isobutyl acetate | 0.14 | 0.14 | 0.14 | 0.00 | 0.00 | 0.00 | 0.19 | 0.15 | 0.22 | 0.39 | 0.33 | 0.43 | 0.31 | 0.33 | 0.27 |
| Ethyl butyrate | 0.00 | 0.00 | 0.00 | 0.77 | 0.71 | 0.73 | 0.00 | 0.00 | 0.00 | 1.35 | 1.31 | 1.01 | 0.66 | 0.62 | 0.64 |
| Butanoic acid, 2-methyl-, ethyl ester | 0.90 | 1.03 | 0.96 | 2.72 | 2.82 | 2.61 | 1.44 | 1.55 | 1.44 | 4.01 | 3.26 | 3.50 | 2.22 | 2.13 | 2.10 |
| Butanoic acid, 3-methyl-, ethyl ester | 0.27 | 0.27 | 0.28 | 0.99 | 1.01 | 0.80 | 0.51 | 0.52 | 0.46 | 1.44 | 1.38 | 1.59 | 1.18 | 1.22 | 1.01 |
| 1,3-Benzenediol, monobenzoate | 0.00 | 0.00 | 0.00 | 2.11 | 1.84 | 1.91 | 0.11 | 0.18 | 0.24 | 0.00 | 0.00 | 0.00 | 0.22 | 0.19 | 0.22 |
| Methyl myristate | 1.16 | 1.28 | 1.01 | 0.66 | 0.56 | 0.59 | 0.88 | 0.83 | 0.76 | 0.55 | 0.58 | 0.55 | 0.46 | 0.48 | 0.46 |
| Ethyl myristate | 0.71 | 0.82 | 0.55 | 1.22 | 1.22 | 1.26 | 1.01 | 0.99 | 1.05 | 2.01 | 2.04 | 1.95 | 1.69 | 1.81 | 1.46 |
| Ethyl palmitate | 4.73 | 6.50 | 4.71 | 11.47 | 14.35 | 9.29 | 13.04 | 11.92 | 6.31 | 0.00 | 0.00 | 0.00 | 0.00 | 0.00 | 0.00 |
| Methyl palmitate | 7.14 | 6.12 | 5.67 | 22.93 | 19.11 | 17.19 | 8.12 | 8.11 | 8.12 | 24.99 | 23.41 | 21.3 | 18.45 | 13.14 | 20.33 |
| Eethyl linoleate | 1.93 | 1.87 | 1.92 | 1.69 | 3.91 | 3.21 | 5.19 | 3.46 | 3.69 | 2.49 | 2.09 | 2.23 | 2.71 | 2.54 | 2.65 |
| Methyl laurate | 0.40 | 0.43 | 0.38 | 0.38 | 0.33 | 0.42 | 0.53 | 0.55 | 0.55 | 0.84 | 0.88 | 0.78 | 1.23 | 1.33 | 1.34 |
| Ethyl laurate | 0.00 | 0.00 | 0.00 | 2.61 | 2.67 | 2.66 | 0.00 | 0.00 | 0.00 | 3.71 | 3.61 | 3.33 | 3.28 | 3.38 | 3.40 |
| Ethyl linoleate | 8.80 | 9.11 | 8.62 | 4.80 | 4.93 | 4.71 | 2.89 | 2.61 | 2.78 | 6.29 | 7.07 | 7.15 | 7.50 | 8.04 | 7.41 |
| Ethyl oleate | 0.68 | 0.70 | 0.61 | 2.81 | 2.44 | 2.69 | 1.29 | 1.47 | 1.40 | 3.29 | 3.88 | 3.49 | 3.29 | 3.33 | 3.42 |
| Methyl acetate | 5.11 | 5.10 | 4.99 | 6.76 | 5.98 | 5.76 | 6.33 | 6.41 | 6.44 | 4.04 | 4.11 | 3.74 | 0.99 | 0.91 | 0.93 |
| Ethyl benzoate | 1.29 | 1.45 | 1.49 | 2.99 | 3.49 | 1.62 | 3.25 | 4.78 | 0.52 | 1.79 | 0.12 | 2.83 | 1.84 | 1.97 | 3.36 |
| Ethyl acetate | 47.73 | 36.22 | 37.64 | 104.28 | 110.49 | 75.88 | 45.98 | 48.87 | 42.67 | 133.91 | 99.47 | 106.98 | 111.38 | 121.83 | 80.07 |
| **Total** | 88.35 | 77.26 | 76.46 | 175.85 | 183.14 | 138.93 | 97.13 | 98.72 | 83.19 | 200.36 | 160.83 | 169.07 | 165.70 | 172.15 | 136.41 |
| **Furan (2 types)** |  |  |  |  |  |  |  |  |  |  |  |  |  |  |  |
| 3-Phenylfuran | 1.77 | 1.80 | 1.60 | 0.45 | 0.45 | 0.46 | 0.49 | 0.48 | 0.48 | 0.77 | 0.77 | 0.70 | 0.00 | 0.00 | 0.00 |
| 3(2H)-Furanone, 4-hydroxy-5-methyl- | 0.00 | 0.00 | 0.00 | 20.21 | 25.03 | 14.10 | 61.94 | 44.86 | 43.88 | 17.99 | 20.14 | 11.12 | 5.30 | 5.12 | 7.04 |
| **Total** | 1.77 | 1.80 | 1.60 | 20.66 | 25.48 | 14.56 | 62.43 | 45.34 | 44.36 | 18.76 | 20.91 | 11.82 | 5.30 | 5.12 | 7.04 |
| **Acid (1 types)** |  |  |  |  |  |  |  |  |  |  |  |  |  |  |  |
| Acetic acid | 59.20 | 64.82 | 46.18 | 90.35 | 75.84 | 66.19 | 55.89 | 61.26 | 44.26 | 44.53 | 39.88 | 39.87 | 70.38 | 64.92 | 45.54 |
| **Total** | 59.20 | 64.82 | 46.18 | 90.35 | 75.84 | 66.19 | 55.89 | 61.26 | 44.26 | 44.53 | 39.88 | 39.87 | 70.38 | 64.92 | 45.54 |
| **Aldehydes (10 types)** |  |  |  |  |  |  |  |  |  |  |  |  |  |  |  |
| Benzene acetaldehyde | 9.58 | 9.20 | 6.95 | 16.57 | 10.29 | 14.60 | 15.62 | 8.00 | 11.64 | 12.79 | 7.12 | 8.96 | 24.66 | 20.11 | 16.11 |
| Benzaldehyde, 2,4-dimethyl- | 1.28 | 1.93 | 1.79 | 2.28 | 2.31 | 2.11 | 2.00 | 2.11 | 1.93 | 2.43 | 2.43 | 2.34 | 2.43 | 2.01 | 1.95 |
| Decanal | 0.00 | 0.00 | 0.00 | 1.20 | 1.24 | 1.17 | 1.25 | 1.10 | 1.11 | 1.39 | 1.42 | 1.28 | 1.58 | 1.21 | 1.23 |
| Benzaldehyde | 14.19 | 17.17 | 10.09 | 23.09 | 31.97 | 20.24 | 20.96 | 15.31 | 17.78 | 24.12 | 24.44 | 16.05 | 34.42 | 32.22 | 22.68 |
| Acetaldehyde | 4.94 | 4.77 | 4.39 | 10.99 | 11.2 | 8.02 | 5.57 | 5.64 | 5.45 | 7.77 | 7.72 | 7.22 | 7.31 | 7.31 | 7.25 |
| 2-Butenal | 0.41 | 0.40 | 0.40 | 1.81 | 1.77 | 1.22 | 0.00 | 0.00 | 0.00 | 1.22 | 1.02 | 1.10 | 0.00 | 0.00 | 0.00 |
| 2-Methylbutyraldehyde | 40.01 | 29.62 | 34.21 | 41.13 | 29.82 | 42.14 | 52.13 | 44.29 | 33.35 | 26.62 | 26.99 | 20.28 | 39.08 | 31.86 | 41.41 |
| 3-Methylbutyraldehyde | 50.79 | 59.13 | 38.56 | 64.16 | 62.37 | 51.64 | 66.58 | 49.77 | 56.51 | 36.61 | 39.37 | 32.54 | 61.17 | 63.00 | 46.30 |
| 3-Furaldehyde | 3.15 | 2.38 | 2.52 | 6.27 | 5.27 | 5.49 | 4.91 | 3.91 | 3.31 | 3.68 | 4.77 | 3.33 | 4.19 | 2.63 | 2.46 |
| Hexanal | 0.61 | 0.93 | 0.69 | 1.02 | 1.09 | 0.88 | 1.23 | 1.42 | 1.81 | 0.81 | 0.88 | 0.71 | 0.58 | 0.56 | 0.59 |
| **Total** | 124.96 | 120.01 | 105.11 | 168.52 | 157.33 | 147.51 | 170.25 | 131.5 | 132.89 | 117.44 | 116.16 | 93.81 | 175.42 | 160.91 | 139.98 |
| **Ketones (7 types)** |  |  |  |  |  |  |  |  |  |  |  |  |  |  |  |
| Methyl Heptenone | 1.47 | 1.87 | 1.33 | 1.77 | 1.51 | 1.49 | 2.39 | 2.03 | 1.76 | 1.66 | 1.63 | 1.51 | 1.32 | 1.30 | 1.19 |
| 5-Ethyl-4-hydroxy-2-methyl-3(2H)-furanone | 0.00 | 0.00 | 0.00 | 17.34 | 14.88 | 17.04 | 5.76 | 5.63 | 6.07 | 20.39 | 19.33 | 19.61 | 61.32 | 47.14 | 42.24 |
| 1-Phenyl-2-butanone | 0.13 | 0.12 | 0.13 | 0.22 | 0.22 | 0.21 | 0.00 | 0.00 | 0.00 | 0.00 | 0.00 | 0.00 | 0.39 | 0.39 | 0.38 |
| Acetone | 2.39 | 1.62 | 2.32 | 2.29 | 2.25 | 2.29 | 2.22 | 2.35 | 2.46 | 1.00 | 1.09 | 1.12 | 1.52 | 1.58 | 1.52 |
| Hydroxyacetone | 0.19 | 0.38 | 0.28 | 0.10 | 0.11 | 0.10 | 0.24 | 0.25 | 0.29 | 0.00 | 0.00 | 0.00 | 0.66 | 0.65 | 0.61 |
| 2-Butanone | 1.55 | 1.8 | 1.34 | 1.88 | 1.81 | 1.72 | 1.62 | 1.67 | 1.58 | 1.22 | 1.35 | 1.01 | 1.85 | 1.83 | 1.73 |
| 1-Octen-3-one | 1.75 | 1.89 | 1.88 | 2.78 | 2.66 | 2.57 | 2.33 | 2.41 | 2.35 | 2.88 | 2.34 | 3.39 | 4.39 | 4.77 | 5.03 |
| **Total** | 7.48 | 7.68 | 7.28 | 26.38 | 23.44 | 25.42 | 14.56 | 14.34 | 14.51 | 27.15 | 25.74 | 26.64 | 71.45 | 57.66 | 52.7 |
| **Phenols (3 types)** |  |  |  |  |  |  |  |  |  |  |  |  |  |  |  |
| 2-Methoxy-4-vinylphenol | 11.31 | 11.73 | 9.42 | 16.13 | 15.11 | 14.2 | 14.36 | 13.74 | 13.69 | 10.28 | 11.06 | 8.13 | 2.98 | 2.32 | 3.12 |
| 2,4-Di-tert-butylphenol | 26.03 | 29.11 | 20.28 | 38.22 | 31.83 | 35.5 | 39.2 | 41.34 | 33.58 | 43.13 | 40.11 | 37.13 | 40.91 | 36.99 | 32.95 |
| Phenol, 2-methoxy- | 0.53 | 0.44 | 0.58 | 0.56 | 0.55 | 0.58 | 0.92 | 0.89 | 0.64 | 1.29 | 1.11 | 0.91 | 1.72 | 1.27 | 1.19 |
| **Total** | 37.87 | 41.28 | 30.28 | 54.91 | 47.49 | 50.28 | 54.48 | 55.97 | 47.91 | 54.70 | 52.28 | 46.17 | 45.61 | 40.58 | 37.26 |
| **Others (4 types)** |  |  |  |  |  |  |  |  |  |  |  |  |  |  |  |
| 2-Hexene, 3,5,5-trimethyl- | 1.89 | 1.81 | 1.54 | 1.33 | 1.38 | 1.41 | 2.01 | 2.07 | 1.93 | 1.44 | 1.63 | 1.39 | 0.00 | 0.00 | 0.00 |
| 2-Pentene, 3-ethyl-2-methyl- | 0.59 | 0.58 | 0.51 | 1.49 | 1.37 | 1.48 | 1.11 | 1.02 | 0.91 | 2.04 | 2.11 | 1.96 | 1.44 | 1.39 | 1.45 |
| 1-Propanol, 3-(methylthio)- | 3.49 | 2.99 | 3.38 | 5.52 | 5.11 | 5.57 | 5.33 | 5.41 | 6.13 | 5.38 | 5.82 | 5.86 | 18.93 | 17.66 | 16.83 |
| Dimethyl trisulfide | 0.19 | 0.18 | 0.19 | 0.00 | 0.00 | 0.00 | 0.19 | 0.19 | 0.29 | 4.34 | 4.28 | 4.56 | 0.22 | 0.21 | 0.23 |
| **Total** | 6.16 | 5.56 | 5.62 | 8.34 | 7.86 | 8.46 | 8.64 | 8.69 | 9.26 | 13.20 | 13.84 | 13.77 | 20.59 | 19.26 | 18.51 |

**Table 12. Means and standard deviations of volatile compounds of five groups of moromi during fermentation (MT: 30°C; LT: 15°C; *S. lloydii*: inoculated *S. lloydii*, 15°C; *L. lactis*: inoculated *L. lactis*, 15°C; *K. ohmeri*: inoculated *K. ohmeri*, 15°C).**

| **Compounds** | | **Content of volatile compounds (μg/L)** | | | | | | | | |  |  |  |  |  |
| --- | --- | --- | --- | --- | --- | --- | --- | --- | --- | --- | --- | --- | --- | --- | --- |
|  |  | **LT** | | ***K. ohmeri*** | | ***L. lactis*** | | ***S. lloydii*** | | **MT** |  |  |  |  |  |
| **Alcohol (14 types)** | |  | |  | |  | |  | |  |  |  |  |  |  |
| Methanethiol | | 0.20±0.07 | | 0.24±0.03 | | 0.22±0.01 | | 0.00±0.00 | | 0.32±0.01 |  |  |  |  |  |
| 2-Naphthalenethiol | | 0.33±0.06 | | 0.60±0.06 | | 0.00±0.00 | | 0.00±0.00 | | 0.39±0.06 |  |  |  |  |  |
| 1-Dodecanol | | 0.00±0.00 | | 0.96±0.10 | | 3.47±0.31 | | 1.12±0.11 | | 0.00±0.00 |  |  |  |  |  |
| Ethanol | | 361.23±25.04 | | 1005.15±11.15 | | 493.18±15.01 | | 1037.58±33.62 | | 1087.08±52.73 |  |  |  |  |  |
| 1-Propanol | | 3.24±0.24 | | 5.40±0.94 | | 5.62±1.31 | | 5.69±1.01 | | 17.81±2.55 |  |  |  |  |  |
| 1-Propanol, 2-methyl- | | 9.15±0.20 | | 17.71±1.67 | | 9.00±0.12 | | 19.39±1.71 | | 11.5±0.23 |  |  |  |  |  |
| 1-Butanol | | 0.28±0.10 | | 2.03±0.09 | | 0.52±0.03 | | 4.04±0.05 | | 5.28±0.06 |  |  |  |  |  |
| Propanal, 2-methyl- | | 2.67±0.29 | | 3.72±0.49 | | 1.30±0.11 | | 1.24±0.06 | | 5.38±0.06 |  |  |  |  |  |
| 1-Octen-3-ol | | 36.24±4.41 | | 47.15±0.00 | | 39.19±0.01 | | 65.20±0.01 | | 14.58±2.57 |  |  |  |  |  |
| 1-Hexanol, 2-ethyl- | | 9.19±0.97 | | 11.49±1.19 | | 11.94±0.04 | | 12.80±0.17 | | 8.21±0.19 |  |  |  |  |  |
| Phenylethyl Alcohol | | 7.85±2.21 | | 16.05±2.05 | | 12.47±3.84 | | 25.99±6.97 | | 65.67±8.99 |  |  |  |  |  |
| 3-Methyl-1-butanol | | 40.48±6.85 | | 57.26±8.91 | | 33.35±7.43 | | 74.80±21.37 | | 83.17±14.36 |  |  |  |  |  |
| 1-Hexanol | | 1.59±0.28 | | 2.28±0.12 | | 2.22±0.08 | | 2.48±0.26 | | 1.16±0.03 |  |  |  |  |  |
| Maltol | | 2.72±0.07 | | 6.99±0.97 | | 5.23±1.81 | | 4.86±1.52 | | 9.53±1.86 |  |  |  |  |  |
| **Total** | | 475.17±25.28 | | 1177.03±19.77 | | 617.71±9.41 | | 1255.19±57.08 | | 1310.17±62.22 |  |  |  |  |  |
| **Esters (23 types)** | |  | |  | |  | |  | |  |  |  |  |  |  |
| Ethyl propionate | | 0.27±0.01 | | 0.60±0.06 | | 0.35±0.03 | | 0.82±0.17 | | 1.23±0.09 |  |  |  |  |  |
| Methyl butyrate | | 1.39±0.11 | | 0.67±0.02 | | 1.17±0.08 | | 0.44±0.03 | | 1.15±0.11 |  |  |  |  |  |
| Ethyl 3-methylbutyrate | | 1.59±0.61 | | 1.09±0.01 | | 1.94±0.06 | | 0.87±0.10 | | 0.55±0.05 |  |  |  |  |  |
| Methyl hexanoate | | 1.14±0.15 | | 1.18±0.16 | | 1.42±0.01 | | 1.23±0.09 | | 0.86±0.02 |  |  |  |  |  |
| Ethyl hexanoate | | 2.22±0.41 | | 1.84±0.03 | | 0.78±0.01 | | 2.51±0.17 | | 0.45±0.01 |  |  |  |  |  |
| Ethyl phenylacetate | | 0.41±0.02 | | 1.80±0.51 | | 0.73±0.01 | | 2.38±0.73 | | 3.96±0.56 |  |  |  |  |  |
| Isobutyl acetate | | 0.14±0.00 | | 0.00±0.00 | | 0.19±0.04 | | 0.38±0.05 | | 0.30±0.03 |  |  |  |  |  |
| Ethyl butyrate | | 0.00±0.00 | | 0.74±0.03 | | 0.00±0.00 | | 1.22±0.19 | | 0.64±0.02 |  |  |  |  |  |
| Butanoic acid, 2-methyl-, ethyl ester | | 0.96±0.07 | | 2.72±0.11 | | 1.48±0.06 | | 3.59±0.38 | | 2.15±0.06 |  |  |  |  |  |
| Butanoic acid, 3-methyl-, ethyl ester | | 0.27±0.01 | | 0.93±0.12 | | 0.50±0.03 | | 1.47±0.11 | | 1.14±0.11 |  |  |  |  |  |
| 1,3-Benzenediol, monobenzoate | | 0.00±0.00 | | 1.95±0.14 | | 0.18±0.07 | | 0.00±0.00 | | 0.21±0.02 |  |  |  |  |  |
| Methyl myristate | | 1.15±0.14 | | 0.60±0.05 | | 0.82±0.06 | | 0.56±0.02 | | 0.47±0.01 |  |  |  |  |  |
| Ethyl myristate | | 0.69±0.14 | | 1.23±0.02 | | 1.02±0.03 | | 2.00±0.05 | | 1.65±0.18 |  |  |  |  |  |
| Ethyl palmitate | | 5.31±1.03 | | 11.70±2.54 | | 10.42±3.61 | | 0.00±0.00 | | 0.00±0.00 |  |  |  |  |  |
| Methyl palmitate | | 6.31±0.75 | | 19.74±2.92 | | 8.12±0.01 | | 23.23±1.85 | | 17.33±3.73 |  |  |  |  |  |
| Ethyl linoleate | | 1.91±0.03 | | 2.93±1.13 | | 4.11±0.94 | | 2.27±0.20 | | 2.63±0.09 |  |  |  |  |  |
| Methyl laurate | | 0.40±0.03 | | 0.38±0.05 | | 0.54±0.01 | | 0.83±0.05 | | 1.30±0.06 |  |  |  |  |  |
| Ethyl laurate | | 0.00±0.00 | | 2.65±0.03 | | 0.00±0.00 | | 3.55±0.20 | | 3.35±0.06 |  |  |  |  |  |
| Ethyl linoleate | | 8.84±0.25 | | 4.81±0.11 | | 2.76±0.14 | | 6.84±0.48 | | 7.65±0.34 |  |  |  |  |  |
| Ethyl oleate | | 0.66±0.05 | | 2.65±0.19 | | 1.39±0.09 | | 3.55±0.30 | | 3.35±0.07 |  |  |  |  |  |
| Methyl acetate | | 5.07±0.07 | | 6.17±0.53 | | 6.39±0.06 | | 3.96±0.20 | | 0.94±0.04 |  |  |  |  |  |
| Ethyl benzoate | | 1.41±0.11 | | 2.70±0.97 | | 2.85±2.16 | | 1.58±1.37 | | 2.39±0.84 |  |  |  |  |  |
| Ethyl acetate | | 40.53±6.28 | | 96.88±18.46 | | 45.84±3.10 | | 113.45±18.11 | | 104.43±21.73 |  |  |  |  |  |
| **Total** | | 80.67±6.65 | | 165.94±23.70 | | 93.00±8.54 | | 176.73±20.85 | | 158.13±19.05 |  |  |  |  |  |
| **Furan (2 types)** | |  | |  | |  | |  | |  |  |  |  |  |  |
| 3-Phenylfuran | | 1.72±0.11 | | 0.45±0.01 | | 0.48±0.01 | | 0.75±0.04 | | 0.00±0.00 |  |  |  |  |  |
| 3(2H)-Furanone, 4-hydroxy-5-methyl- | | 0.00±0.00 | | 19.78±5.48 | | 50.23±10.16 | | 16.42±4.71 | | 5.82±1.06 |  |  |  |  |  |
| **Total** | | 1.72±0.11 | | 20.23±5.47 | | 50.71±10.16 | | 17.17±4.75 | | 5.82±1.06 |  |  |  |  |  |
| **Acid (1 types)** | |  | |  | |  | |  | |  |  |  |  |  |  |
| Acetic acid | | 56.73±9.56 | | 74.46±12.26 | | 53.80±8.96 | | 41.43±2.69 | | 60.28±13.05 |  |  |  |  |  |
| **Total** | | 56.73±9.56 | | 74.46±12.26 | | 53.80±8.96 | | 41.43±2.69 | | 60.28±13.05 |  |  |  |  |  |
| **Aldehydes (10 types)** | |  | |  | |  | |  | |  |  |  |  |  |  |
| Benzene acetaldehyde | | 8.58±1.42 | | 13.82±3.21 | | 11.74±3.84 | | 9.62±2.89 | | 20.29±4.28 |  |  |  |  |  |
| Benzaldehyde, 2,4-dimethyl- | | 1.67±0.34 | | 2.23±0.11 | | 2.01±0.09 | | 2.40±0.05 | | 2.13±0.26 |  |  |  |  |  |
| Decanal | | 0.00±0.00 | | 1.20±0.04 | | 1.15±0.08 | | 1.36±0.07 | | 1.34±0.21 |  |  |  |  |  |
| Benzaldehyde | | 13.82±3.55 | | 25.10±6.12 | | 18.02±2.83 | | 21.55±4.75 | | 29.77±6.24 |  |  |  |  |  |
| Acetaldehyde | | 4.70±0.28 | | 10.07±1.78 | | 5.55±0.10 | | 7.57±0.30 | | 7.29±0.03 |  |  |  |  |  |
| 2-Butenal | | 0.40±0.01 | | 1.60±0.33 | | 0.00±0.00 | | 1.11±0.10 | | 0.00±0.00 |  |  |  |  |  |
| 2-Methylbutyraldehyde | | 34.61±5.21 | | 37.70±6.84 | | 43.26±9.43 | | 24.63±3.77 | | 37.45±4.98 |  |  |  |  |  |
| 3-Methylbutyraldehyde | | 49.49±10.35 | | 59.39±6.77 | | 57.62±8.46 | | 36.17±3.44 | | 56.82±9.16 |  |  |  |  |  |
| 3-Furaldehyde | | 2.68±0.41 | | 5.68±0.53 | | 4.04±0.81 | | 3.93±0.75 | | 3.09±0.95 |  |  |  |  |  |
| Hexanal | | 0.74±0.17 | | 1.00±0.11 | | 1.49±0.30 | | 0.80±0.09 | | 0.58±0.02 |  |  |  |  |  |
| **Total** | | 116.69±14.81 | | 157.80±10.51 | | 144.88±21.98 | | 109.15±13.29 | | 158.77±17.82 |  |  |  |  |  |
| **Ketones (7 types)** | |  | |  | |  | |  | |  |  |  |  |  |  |
| Methyl Heptenone | | 1.56±0.28 | | 1.59±0.61 | | 2.06±0.32 | | 1.60±0.08 | | 1.27±0.07 |  |  |  |  |  |
| 5-Ethyl-4-hydroxy-2-methyl-3(2H)-furanone | | 0.00±0.00 | | 16.42±1.34 | | 5.82±0.23 | | 19.78±0.55 | | 50.23±9.91 |  |  |  |  |  |
| 1-Phenyl-2-butanone | | 0.13±0.01 | | 0.22±0.01 | | 0.00±0.00 | | 0.00±0.00 | | 0.39±0.01 |  |  |  |  |  |
| Acetone | | 2.11±0.43 | | 2.28±0.02 | | 2.34±0.12 | | 1.07±0.06 | | 1.54±0.03 |  |  |  |  |  |
| Hydroxyacetone | | | 0.28±0.10 | | 0.10±0.01 | | 0.26±0.03 | | 0.00±0.00 | | 0.64±0.03 |  |  |  |  |
| 2-Butanone | | | 1.56±0.23 | | 1.80±0.08 | | 1.62±0.05 | | 1.19±0.17 | | 1.80±0.06 |  |  |  |  |
| 1-Octen-3-one | | | 1.84±0.08 | | 2.67±0.11 | | 2.36±0.04 | | 2.87±0.53 | | 4.73±0.32 |  |  |  |  |
| **Total** | | | 7.49±1.20 | | 25.09±1.50 | | 14.46±0.12 | | 26.51±0.71 | | 60.61±9.72 |  |  |  |  |
| **Phenols (3 types)** | |  | |  | |  | |  | |  |  |  |  |  |  |
| 2-Methoxy-4-vinylphenol | | | | 10.82±1.23 | | 15.15±0.97 | | 13.93±0.37 | | 9.82±1.52 | | 2.81±0.43 | |  |  |
| 2,4-Di-tert-butylphenol | | | | 25.14±4.48 | | 35.18±3.21 | | 38.04±4.01 | | 40.12±3.00 | | 36.95±3.98 | |  |  |
| Phenol, 2-methoxy- | | | | 0.52±0.07 | | 0.56±0.02 | | 0.82±0.15 | | 1.10±0.19 | | 1.39±0.29 | |  |  |
| **Total** | | | | | | 36.47±5.63 | | 50.89±3.75 | | 52.79±4.29 | | 51.04±4.40 | | 41.14±4.20 |  |
| **Others (4 types)** | | |  | |  | |  | |  | |  | |  |  |  |
| 2-Hexene, 3,5,5-trimethyl- | | | 1.75±0.18 | | 1.37±0.04 | | 2.00±0.07 | | 1.49±0.13 | | 0.00±0.00 | |  |  |  |
| 2-Pentene, 3-ethyl-2-methyl- | | | 0.56±0.04 | | 1.45±0.07 | | 1.01±0.10 | | 2.04±0.08 | | 1.43±0.03 | |  |  |  |
| 1-Propanol, 3-(methylthio)- | | | | | | 3.29±0.26 | | 5.40±0.25 | | 5.62±0.44 | | 5.69±0.27 | | 17.81±1.06 | |
| Dimethyl trisulfide | | | | | | 0.19±0.01 | | 0.00±0.00 | | 0.22±0.06 | | 4.39±0.15 | | 0.22±0.01 | |
| **Total** | | | | 5.79±0.33 | | 8.22±0.32 | | 8.84±0.34 | | 13.62±0.35 | | 19.46±1.05 | |  |  |
| **All compounds total** | | | | 780.73±50.87 | | 1661.74±64.82 | | 987.37±35.57 | | 1676.01±89.01 | | 1810.00±83.33 | |  |  |
