# Supplementary figures and images for: Simulate the natural four-season fermentation system for high-salt diluted-state soy sauce brewing: Application in flavor promotion regulation
Source: PLoS One. 2025 Oct 16;20(10):e0334707. doi: 10.1371/journal.pone.0334707 (PMC12530599; doi:10.1371/journal.pone.0334707)

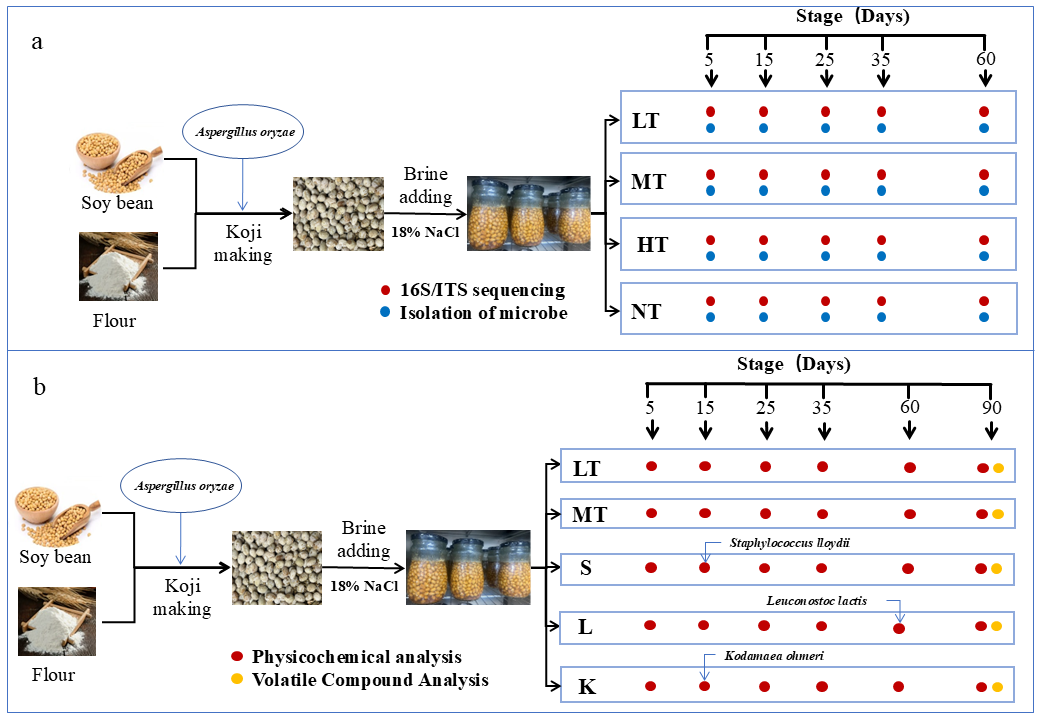

Supplement: S2 File — Schematic diagram of the laboratory-scale simulated fermentation system, sample collection, and experimental design. (a) 60 moromi samples were separately collected from three batches of LT, MT, HT, and NT on the 5th, 15th, 25th, 35th, and 60th day of fermentation. Schematic of the backfilling experimental design with collected sample and methodology types; (b) 90 moromi samples were collected from three batches of LT, MT, HT and NT at 5th, 15th, 25th, 35th, 60th and 90th day of fermentation (HT: 37°C; MT: 30°C; NT: 25°C; LT: 15°C; S: inoculated S. lloydii, 15°C; L: inoculated L. lactis, 15°C; S: inoculated K. ohmeri, 15°C). Points of different shapes represent different analysis methods used. (TIF) [file pone.0334707.s002.TIF]
